# Machine-learning prediction of pre-dose pharmacokinetics optimizes initial vancomycin dosing in critically ill children

**DOI:** 10.3389/fphar.2026.1839423

**Published:** 2026-05-26

**Authors:** Jihui Chen, Libo Dai, Haixin Xu, Yunlu Yao, Xiaohui Huang, Jing Ma, Xinzhu Liu, Huijuan Yao, Jiru Li, Dan Wu, Jincheng Sun, Ze Yu, Yanhui Li, Hongxin Yang, Shuhong Bu

**Affiliations:** 1 Department of Clinical Pharmacy, Xinhua Hospital, School of Medicine, Shanghai Jiao Tong University, Shanghai, China; 2 Department of Clinical Pharmacy, Inner Mongolia Autonomous Region People’s Hospital, Hohhot, China; 3 College of Pharmacy, Dalian Medical University, Dalian, China; 4 Department of Pediatric Critical Care Medicine, Xinhua Hospital, School of Medicine, Shanghai Jiao Tong University, Shanghai, China; 5 Beijing Medicinovo Technology Co. Ltd., Beijing, China; 6 Inner Mongolia Research Institute, Shanghai Jiao Tong University, Hohhot, China

**Keywords:** initial dosing, machine learning, model-informed precision dosing, population pharmacokinetics, vancomycin

## Abstract

**Objectives:**

To develop and validate a machine-learning/population-pharmacokinetic (ML-PPK) hybrid model that predicts individual vancomycin clearance (CL) and volume of distribution (V_d_) before the first dose, thereby informing initial dosing in critically ill children.

**Patients and methods:**

We retrospectively analysed children from two tertiary centers in China (2013–2023). A previously published one-compartment PPK model was re-estimated with the pooled dataset and used as a Bayesian prior to derive individual CL and V_d_ as training targets. Ten machine-learning and deep-learning algorithms were trained, and an XGBoost-based sequential forward-selection procedure was applied to identify a minimal predictor set. Model performance was evaluated on a held-out test set and an external cohort.

**Results:**

Data from 821 children and 1,767 vancomycin concentrations were included. 29 candidate variables were screened, and six high-impact predictors - body weight, cardiothoracic surgery, estimated glomerular filtration rate, sex, ICU admission, and post-menstrual-age class - maximized performance. CatBoost outperformed the other evaluated algorithms and, under this study design, more closely approximated PPK-Bayesian posterior PK estimates than the original parametric PPK covariate model, yielding for CL: R^2^ = 0.89, and 81.8% of predictions within ±30%; and for V_d_: R^2^ = 0.95, with 92.1% within ±30%. Performance remained robust in both the test set and the external validation cohort, with external validation R^2^ values of 0.85 for CL and 0.95 for V_d_. SHAP analysis highlighted body weight, renal function, and cardiothoracic surgery status as the main determinants of CL, consistent with covariate effects in the PPK model.

**Conclusion:**

An interpretable CatBoost-based ML-PPK hybrid can estimate CL and V_d_ pre-dose using routinely available data, enabling patient-specific initial vancomycin regimens and reducing early under- or overexposure in pediatric critical care.

## Introduction

Vancomycin remains a first-line antimicrobial agent for treating methicillin-resistant *Staphylococcus aureus* (MRSA) infections. Its clinical use is further complicated by a narrow therapeutic index and marked inter-individual pharmacokinetic variability, particularly among critically ill children (Aljutayli et al., 2022; Chuphan et al., 2022). Accordingly, therapeutic drug monitoring (TDM) has become an integral component of vancomycin therapy, allowing clinicians to optimize exposure while limiting toxicity (Matsumoto et al., 2022). Current guidelines recommend targeting the area under the concentration-time curve over 24 h (AUC_24_) as superior to trough concentration monitoring for optimizing vancomycin therapy (He et al., 2020; Rybak et al., 2020). Population pharmacokinetic (PPK) models, often integrated with Bayesian forecasting, enable estimation of vancomycin AUC_24_ and guide subsequent dose adjustments during TDM (De Velde et al., 2018). While guideline-endorsed and effective for post-TDM dose optimization, this approach remains inadequate for individualizing the initial vancomycin dose - especially in critically ill children (Sosnin et al., 2019).

Determining an appropriate initial vancomycin regimen remains a significant clinical challenge in children with severe infections. Subtherapeutic concentrations due to underdosing compromise early therapeutic efficacy, potentially delaying infection control (Hsu et al., 2018). Dose adjustments based on initial TDM typically require 2–3 days, resulting in a critical window where treatment may be ineffective. Conversely, excessively high doses elevate the risk of acute kidney injury (AKI), which can develop rapidly in critically ill patients before the first TDM results are available (Le et al., 2015). Early exposure is pivotal, yet most centers still rely on weight-based empirical protocols because a robust, patient-specific method for selecting the initial dose is lacking. Conventional PPK models typically incorporate key covariates such as body weight, age or postmenstrual age, and renal function, which have consistently been identified as major determinants of vancomycin pharmacokinetics in pediatric populations (Chung et al., 2021). However, their predictive performance is often still inadequate for individualized initial dosing (Mena et al., 2023). More advanced approaches are therefore needed to improve predictive accuracy.

Advancements in artificial intelligence have driven increased interest in machine learning (ML) for predictive modeling in pharmacology. Previous research, including our own, indicates ML models can outperform traditional PPK models in predicting effective vancomycin initial doses (Huang et al., 2021; Lee et al., 2025). However, a significant limitation of ML is its inherent “black box” nature, often yielding predictions without transparent, clinically interpretable rationale, thereby hindering clinical adoption. PPK models, in contrast, leverage mechanism-based pharmacokinetic principles within statistical frameworks, offering greater interpretability. Combining ML’s predictive power with PPK’s mechanistic foundation in a hybrid model presents a promising strategy to enhance both predictive accuracy and model interpretability.

Preliminary studies have explored ML–PPK hybrid models for vancomycin, but these have mostly focused on predicting single drug concentrations and often rely on high-dimensional inputs with “black-box” outputs, which limits clinician trust and bedside use (Chen et al., 2025; Yu et al., 2025). To move beyond this, we chose to predict complete pharmacokinetic (PK) parameters. This strategy offers several advantages: it allows direct calculation of steady-state AUC and enables simulation of concentration–time profiles at any time point, thereby markedly improving clinical utility. In parallel, current model-informed precision dosing (MIPD) practice relies on PPK–Bayesian tools that are well established for post-TDM dose adjustment, yet there is still no widely adopted, accurate method for pre-dose individualization of the initial vancomycin regimen in critically ill children. Accordingly, we developed an ML–PPK hybrid model and compared its performance with the original parametric PPK covariate model for estimating vancomycin PK parameters without prior concentration data in a cohort of critically ill children. Our aim was twofold: (i) to address the unmet need for accurate, patient-specific initial dosing, and (ii) to demonstrate that an interpretable, low-dimensional ML–PPK hybrid can improve predictive performance without increasing clinical complexity.

## Methods

### Study design and patients

This retrospective two-center study reviewed electronic medical records from two tertiary institutions: Xinhua Hospital, Shanghai Jiao Tong University School of Medicine (June 2013 – March 2023) and the People’s Hospital of the Inner Mongolia Autonomous Region (June 2018 – March 2023). Children (<18 years) were eligible if they were classified as critically ill, and had received vancomycin for ≥3 consecutive days, and had at least one serum concentration measurement. Critically ill status was defined as suspected or proven infection plus a pSOFA score ≥2. Patients undergoing renal replacement therapy or extracorporeal membrane oxygenation were excluded. For external validation, additional children from Shanghai Children’s Medical Center were included.

### Data collection and preprocessing

According to clinical experience and previous literature, we retrospectively retrieved clinical and laboratory data from electronic medical records, including demographic factors (age, postmenstrual age [PMA], height, weight, sex), hospitalization and surgical information (ICU, after cardiac surgery [CTS]), Laboratory parameters. We defined CTS patients as those who underwent CTS and received concomitant vasoactive agents (including milrinone, dopamine, epinephrine or norepinephrine) during vancomycin therapy, to better capture children in the early postoperative period with ongoing hemodynamic instability, and CTS status was handled as a time-varying covariate in the PPK model.

Vancomycin was administered via intermittent IV infusion, with the typical infusion duration being either 1 or 3 h. Blood samples were collected as part of routine TDM procedures. At steady state, peak concentrations were typically drawn within 1 h after the end of infusion, while trough concentrations were generally obtained within 30 min before the next scheduled dose. Serum vancomycin concentrations were analyzed using HPLC, as described previously (Huang et al., 2021).

Estimated glomerular filtration rate (eGFR, mL/min/1.73 m^2^) was calculated using the same equation as in our previous pediatric vancomycin PPK model (Schwartz et al., 2009; Chen et al., 2023):

$$\mathrm{e}GFR={\;40.7\times \left(\frac{h\mathrm{e}\mathrm{i}ght\;\left(m\right)}{SCr\;\left(mg/dL\right)}\right)}^{0.64}\times {\left(\frac{30}{BUN\;\left(mg/dL\right)}\right)}^{0.202}$$

### Population pharmacokinetic (PPK) analysis

Building on our previously published pediatric one-compartment vancomycin PPK model (Chen et al., 2023), we performed a model-updating procedure in which the original structural and covariate framework was retained and all model parameters were re-estimated using the pooled two-center dataset. Modelling was performed in NONMEM v7.6 (ICON plc, United States) with first-order conditional estimation and interaction (FOCE-I). The updated model was then applied in a Bayesian posterior step to obtain individual pharmacokinetic parameters - clearance (CL) and volume of distribution (V_d_) - for every patient. These patient-specific CL and V_d_ values served as the target variables for subsequent ML model development, because directly observed individual PK parameters were not available in this real-world dataset. Full details of the base model structure, and diagnostic procedures follow the methodology described previously (Chen et al., 2023).

### Feature engineering and model establishment

To enhance the model’s accuracy, the data underwent preprocessing as follows: continuous variables were subjected to a log-log transformation, while binary variables were encoded using one-hot encoding. The Random Forest (RF) algorithm was used to impute missing data.

**FIGURE 1:**
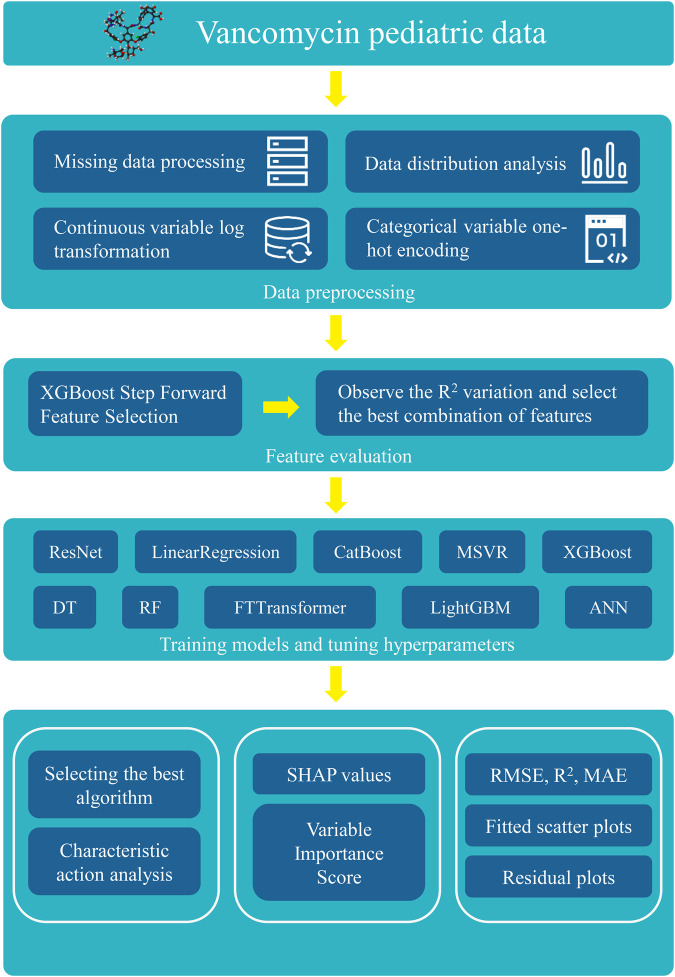
Flowchart of data analysis and model development. Abbreviations: FTTransformer, feature token transformer; RF, random forest; ResNet, residual networks; MSVR, multi-output support vector regression; XGBoost, extreme gradient boosting; DT, decision tree; CatBoost, category-boosted trees; ANN, artificial neural network; LGBM, light gradient boosted machine; R^2^, coefficient of determination.

The workflow of modeling is depicted in Figure 1. After preprocessing, the dataset was randomly divided into training and test sets at an 8:2 ratio, without explicit stratification. For the training set, we performed 10-fold cross-validation and parameter optimization for ten machine-learning and deep-learning models to construct predictive models. Ten machine learning algorithms (Supplementary information), FTTransformer, RF, ResNet, LinearRegression, Multi-output support vector regression (MSVR), extreme gradient boosting (XGBoost), decision tree (DT), categorical boosting (CatBoost), artificial neural network (ANN), light gradient boosting machine (LightGBM), were constructed for the vancomycin CL prediction. Supplementary Tables S1, S2 provide detailed descriptions of the model parameter data, including the parameters selected through 10-fold cross-validation and the optimal parameters for the 10 best-performing models. The analysis code used has been made publicly available at GitHub (https://github.com/RonghongJi/Pedi-Vanco-MTR). The test set serves to assess the model’s performance. This study compared the performance of 10 vancomycin CL prediction models and selected the one with the best indicator values as the optimal prediction model. The performance of the chosen model was assessed using the coefficient of determination (*R* ^2^), root mean squared error (RMSE) and mean absolute error (MAE). The calculation formulas are as follows:

$$R^{2}=1-\frac{\text{MSE}\left(\hat{y},y\right)}{\text{Var}\left(y\right)}$$

$$\text{RMSE}=\sqrt{\frac{1}{n}\sum_{i=1}^{n}{\left(y_{i}-{\hat{y}}_{i}\right)}^{2}}$$

$$\text{MAE}=\frac{1}{n}\sum_{i=1}^{n}\left|\left(y_{i}-{\hat{y}}_{i}\right)\right|$$

The R^2^ indicates the goodness of fit, ranging from 0 to 1, with higher values suggesting better model fit. Lower values of RMSE and MAE indicate superior model performance. Optimal parameter combinations were identified using 80% of the training data, referred to as the analysis subset. Model performance was subsequently evaluated on the held-out test set and in the external validation cohort. To further assess the robustness of the best-performing model, we performed bootstrap resampling (5,000 replicates) on the held-out test set and the external validation cohort.

Moreover, we systematically analyzed the role of individual features in the optimal model and derived their respective importance scores. To further interpret the contributions of these features to the model’s performance, the Shapley Additive exPlanations (SHAP) technique was applied (Lundberg and Lee, 2017).

### Subgroup analysis

To compare ML and PPK performance across clinically relevant strata, we conducted subgroup analyses by developmental stage and renal function: five PMA/age groups (PMA <44 weeks, 44 weeks to <1 year, 1 to <4 years, 4–10 years, ≥10 years) and four eGFR groups (<60, 60–89, 90–119, ≥120 mL/min). Performance in each subgroup was quantified using validation metrics, and prediction accuracy was summarized at ±20% to ±50% thresholds. All PPK-based predictions, including subgroup analyses, were generated using the updated PPK model.

### Statistical analysis

All baseline characteristics were evaluated for their correlation with CL. Data normality was assessed using the Kolmogorov-Smirnov test. For normally distributed data, univariate analyses were performed using t-test for categorical variables and Pearson correlation analysis for continuous variables. For non-normally distributed data, the Mann-Whitney U test was employed for categorical variables, while Spearman correlation analysis was utilized for continuous variables. Statistical analyses were conducted using SPSS (version 25.0) and Python (version 3.7).

## Results

### Baseline information

A total of 852 children were initially screened for eligibility. After excluding 31 children who were receiving renal replacement therapy or extracorporeal membrane oxygenation, 821 children with 1767 vancomycin concentrations were included in the final analysis (Table 1). Of these, 753 were from Xinhua Hospital and 68 were from the People’s Hospital of the Inner Mongolia Autonomous Region. An additional 53 children from Shanghai Children’s Medical Center were included as the external validation cohort. Baseline characteristics across the three centers are provided in Supplementary Table S3. The median (IQR) age of the children was 1.75 (0.41–6.41) years, with a median (IQR) weight of 11.00 (6.00–20.00) kg, and a median (IQR) height of 81.00 (62.00–115.00) cm. In addition, the median (IQR) of PMA was 131.14 (61.43–374.43). After stratifying the PMA class, 9.74% of the values were below 44. In the hospitalization and surgical information, the proportions of CTS and ICU were 10.48% and 82.22% respectively.

**TABLE 1:** Demographic and characteristic statistical description.

Category	Variable	Median (IQR)|n (%)	Miss rate
Target variable	CL, median (IQR)	1.56 (0.69∼3.01)	0.00%
	V_d_, median (IQR)	5.80 (3.22∼10.82)	0.00%
Demographic information	Age, year, median (IQR)	1.75 (0.41∼6.41)	0.00%
	PMA, median (IQR)	131.14 (61.43∼374.43)	0.00%
	PMA_class, n (%)	​	0.00%
	PMA<44 weeks	80 (9.74%)	​
	PMA≥44 weeks	741 (90.26%)	​
	Height, median (IQR)	81.00 (62.00∼115.00)	0.00%
	Weight, median (IQR)	11.00 (6.00∼20.00)	0.00%
	Sex, n (%)	​	0.00%
	Male	478 (58.22%)	​
	Female	343 (41.78%)	​
Hospitalization and surgical information	CTS, n (%)	86 (10.48%)	0.00%
	ICU, n (%)	675 (82.22%)	0.00%
Laboratory parameters	UA (μmol/L), median (IQR)	152.40 (95.50∼233.64)	2.68%
	Scr (μmol/L), median (IQR)	23.80 (17.60∼33.00)	0.00%
	BUN (mmol/L), median (IQR)	3.60 (2.45∼5.00)	0.00%
	eGFR (mL/min), median (IQR)	108.40 (85.17∼120.00)	0.00%
	Hb (g/L), median (IQR)	99.00 (88.00∼111.00)	0.12%
	ALB (g/L), median (IQR)	36.30 (31.60∼40.50)	0.00%
	DBIL (μmol/L), median (IQR)	0.00 (0.00∼1.00)	1.58%
	TBIL (μmol/L), median (IQR)	10.90 (6.00∼20.70)	1.58%
	TP (g/L), median (IQR)	62.00 (55.77∼69.53)	1.58%
	PCT (ng/mL), median (IQR)	0.33 (0.12∼1.55)	7.80%
	ALT (U/L), median (IQR)	29.80 (19.00∼48.30)	1.71%
	AST (U/L), median (IQR)	45.25 (30.00∼82.00)	1.58%
	WBC (10^9^/L), median (IQR)	10.04 (7.06∼14.36)	0.12%
	NEUT (10^9^/L), median (IQR)	5.86 (3.45∼9.80)	0.61%
	LY (10^9^/L), median (IQR)	2.44 (1.37∼3.74)	0.61%
	RBC (10^12^/L), median (IQR)	3.54 (3.13∼3.96)	0.12%
	HCT (%), median (IQR)	29.85 (26.80∼33.50)	0.12%
	PLT (10^9^/L), median (IQR)	285.00 (169.75∼432.00)	0.12%
	CRP (mg/L), median (IQR)	16.27 (8.00∼49.00)	2.19%
Vancomycin concentration observations	1 concentration per patient, n (%)	89 (10.8%)	-
	2 concentrations per patient, n (%)	649 (79.0%)	-
	≥3 concentrations per patient, n (%)	83 (10.1%)	-

Abbreviations: CL, plasma clearance; V_d_, volume; PMA, postmenstrual age; ICU, intensive care unit; CTS, after cardiac surgery; UA, uric acid; Scr, serum creatinine; BUN, blood urea nitrogen; eGFR, estimated glomerular filtration rate; Hb, hemoglobin; ALB, albumin; DBIL, direct bilirubin; TBIL, total bilirubin; TP, total protein; PCT, procalcitonin; ALT, alanine transaminase; AST, aspartate transaminase; WBC, white blood cell; NEUT, neutrophil count; LY, lymphocyte; RBC, red blood cell; HCT, hematocrit; PLT, platelet; CRP, C-reactive protein.

### Individual pharmacokinetic-parameter estimation

To obtain robust PK parameters for every enrolled child, we retained the previously published one-compartment population pharmacokinetic framework and re-estimated its coefficients with the full multicenter dataset (821 children; 1767 vancomycin concentrations). Among the 1767 vancomycin concentrations included in the analysis, 840 were peak concentrations and 927 were trough concentrations. In addition, 732 children (89.2%) contributed at least two concentrations, providing a relatively data-rich basis for Bayesian estimation of individual PK parameters. The updated estimates closely mirrored the original values (Supplementary Table S4), and goodness-of-fit diagnostics demonstrated an excellent match between model predictions and observations (Supplementary Figure S1). In addition, no new covariates, including study center, materially improved the model during updating. Together, these findings support the adequacy and stability of the original structural and covariate framework in the expanded dataset. The final PPK model is as follows:

$$\text{CL}=7.84\times {\left(\frac{\text{WT}}{70}\right)}^{0.75}\times \left[\frac{1}{1+{\left(\frac{\text{PMA}}{37.1}\right)}^{-1.64}}\right]\times {\left(\frac{\text{eGFR}}{109}\right)}^{1.02}\times 0.783^{\text{CTS}}\times e^{\eta_{1}}$$

$$V_{d}=36.6\times \frac{\text{WT}}{70}\times {\left(\frac{36.2}{\text{ALB}}\right)}^{0.255}\times e^{\eta_{2}}$$

WT represents body weight (kg), PMA represents postmenstrual age (weeks), eGFR represents estimated glomerular filtration rate (mL/min), CTS represents cardiothoracic surgery (CTS patients: CTS = 1; non-CTS patients: CTS = 0), and ALB represents albumin (g/L). η_1_ and η_2_ denote interpatient variation.

Using this updated model as the Bayesian prior, we derived individual posterior estimates of CL and V_d_ for all patients (Table 1).

### Variable selection

As shown in Table 1, a total of 29 variables were initially recorded. Then, to optimize the predictive model for vancomycin CL, a forward Sequential Forward Selection (SFS) algorithm based on XGBoost was employed to identify the optimal combination of variables from 1 to 27 potential candidates. With an increasing number of variables included, the model’s *R* ^2^ value improved accordingly, reaching a peak of 0.86 with six variables (Supplementary Figure S2). To achieve a parsimonious and accurate model for optimal predictive performance, six key predictors-weight, CTS, eGFR, sex, ICU, and PMA class-were selected to develop a personalized medication model.

### Machine learning model performance

**FIGURE 2:**
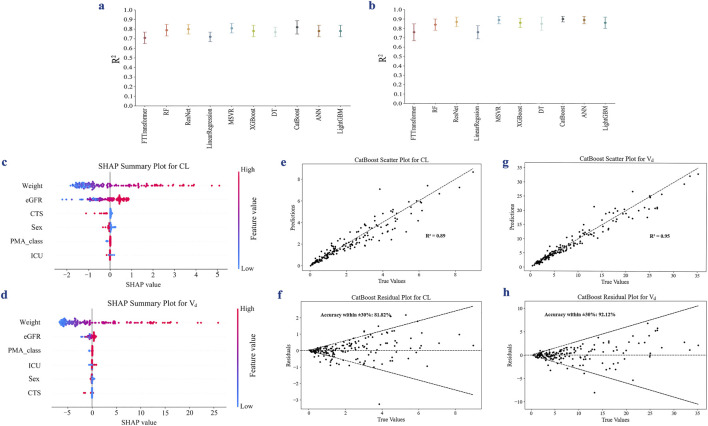
**(a)** The R^2^ (mean ± std) results of ten-fold cross-validation for CL; **(b)** the R^2^ (mean ± std) results of ten-fold cross-validation for V_d_; **(c)** SHAP dependency diagram based on CatBoost model for CL; **(d)** SHAP dependency diagram based on CatBoost model for V_d_; **(e)** scatter plot based on the CatBoost model for CL; **(f)** residual plot based on the CatBoost model for CL; **(g)** scatter plot based on the CatBoost model for V_d_; **(h)** residual plot based on the CatBoost model for V_d_. Abbreviations: CTS, after cardiac surgery; ICU, intensive care unit; eGFR, estimated glomerular filtration rate; PMA, postmenstrual age.

The results of the ten-fold cross-validation for different models are illustrated in Figures 2a,b, and Supplementary Tables S5, S6. From the figure, it can be observed that the CatBoost algorithm achieved the highest performance, with a mean R^2^ of 0.82 (std: 0.07) for CL and a mean R^2^ of 0.90 (std: 0.03) for V_d_. Compared to other models, the CatBoost algorithm demonstrated superior performance. Table 2; Supplementary Figure S3 present the validation results for CL and V_d_ on the test set, respectively. Among the ten algorithms, the CatBoost model achieved the best performance in terms of RMSE, R^2^, and MAE. Specifically, the CatBoost model yielded an RMSE of 0.60, an R^2^ of 0.89, and an MAE of 0.38 for CL; for V_d_, the RMSE was 1.75, the *R* ^2^ was 0.95, and the MAE was 1.12. Overall, the CatBoost algorithm outperformed other models, including the traditional PPK method, and was therefore selected as the final predictive model. The test set accuracy of different models is summarized in Table 3. The CatBoost model achieved an accuracy of 81.8% within ±30% for CL and 92.1% within ±30% for V_d_. In comparison, the PPK model achieved an accuracy of 76.9% within ±30% for CL and 89.9% within ±30% for V_d_. These results indicate that the CatBoost algorithm not only outperformed the PPK model but also exhibited superior performance compared to other models. Bootstrap analysis of the test set further supported the robustness of these findings (Supplementary Table S7). For CL, CatBoost achieved an RMSE of 0.596 (95% CI, 0.471–0.725), an *R* ^2^ of 0.891 (0.828–0.933), and 81.8% accuracy within ±30% (75.8%–87.3%), compared with 0.659 (0.534–0.796), 0.867 (0.794–0.914), and 77.0% (70.3%–83.0%) for the PPK model. The CatBoost–PPK differences excluded zero for RMSE, *R* ^2^, and MAE, but not for ±30% accuracy. For V_d_, the point estimates also favored CatBoost, but the corresponding difference confidence intervals were smaller and less consistent, with the ±30% accuracy difference not excluding zero.

**TABLE 2:** Validation results of CL and V_d_ on the test set.

Algorithm	CL (L/h)			V_d_ (L)		
	RMSE	R^2^	MAE	RMSE	R^2^	MAE
FTTransformer	0.90	0.75	0.60	2.33	0.90	1.46
RF	0.73	0.84	0.54	2.69	0.87	1.93
ResNet	0.86	0.78	0.58	2.80	0.86	1.76
LinearRegression	0.88	0.76	0.67	3.20	0.82	2.45
MSVR	0.70	0.85	0.50	2.14	0.92	1.54
XGBoost	0.73	0.84	0.54	2.56	0.89	1.81
DT	0.70	0.85	0.50	1.97	0.93	1.35
CatBoost	**0.60**	**0.89**	**0.38**	**1.75**	**0.95**	**1.12**
ANN	0.69	0.86	0.52	1.96	0.93	1.35
LightGBM	0.79	0.81	0.57	2.46	0.89	1.75
PPK	0.69	0.87	0.44	1.98	0.93	1.21

Abbreviations: FTTransformer, feature token transformer; RF, random forest; ResNet, residual networks; MSVR, multi-output support vector regression; XGBoost, extreme gradient boosting; DT, decision tree; CatBoost, category-boosted trees; ANN, artificial neural network; LGBM, light gradient boosted machine; R^2^, coefficient of determination; RMSE, root mean square error; MAE, mean absolute error; PPK, population pharmacokinetics. Bold values indicate the best-performing algorithm for the corresponding metric.

**TABLE 3:** Accuracy results of CL and V_d_ on the test set.

Algorithm	CL			V_d_		
	±20%	±30%	±50%	±20%	±30%	±50%
FTTransformer	37.0	56.4	84.9	66.7	90.9	98.8
RF	39.4	59.4	78.2	50.9	64.9	79.4
ResNet	39.4	65.5	91.5	58.2	86.7	98.2
LinearRegression	36.4	54.6	67.9	43.6	54.6	68.5
MSVR	46.1	61.8	74.6	57.6	69.7	86.1
XGBoost	43.0	58.2	74.6	44.2	64.9	76.4
DT	43.3	67.3	84.2	69.1	86.1	95.8
CatBoost	**60.0**	**81.8**	**92.1**	**80.0**	**92.1**	**98.2**
ANN	48.5	59.4	73.3	67.9	81.8	89.1
LightGBM	40.0	55.8	73.3	45.5	65.5	76.4
PPK	54.1	76.9	91.7	75.9	89.9	96.8

Data are presented as percentages (% of patients) within the indicated error range.

Abbreviations: FTTransformer, feature token transformer; RF, random forest; ResNet, residual networks; MSVR, multi-output support vector regression; XGBoost, extreme gradient boosting; DT, decision tree; CatBoost, category-boosted trees; ANN, artificial neural network; LGBM, light gradient boosted machine; PPK, population pharmacokinetics. Bold values indicate the best-performing algorithm for the corresponding metric.

In the external validation cohort (Supplementary Tables S8, S9), CatBoost remained robust, yielding for CL an RMSE of 0.71, R^2^ of 0.85, MAE of 0.39, and 84.9% of predictions within ±30%; the corresponding values for V_d_ were RMSE of 2.38, R^2^ of 0.95, MAE of 1.33, and 100.0% within ±30%. Bootstrap analysis of the external validation cohort showed a similar pattern (Supplementary Table S10). For CL, CatBoost yielded an RMSE of 0.713 (95% CI, 0.373–1.009), an R^2^ of 0.847 (0.644–0.951), and 84.9% accuracy within ±30% (75.4%–94.3%), compared with 0.793 (0.449–1.110), 0.810 (0.593–0.929), and 79.2% (67.9%–88.7%) for the PPK model; among the pairwise differences, only MAE excluded zero. For V_d_, CatBoost achieved an RMSE of 2.378 (1.324–3.382), an R^2^ of 0.947 (0.915–0.979), and 100.0% accuracy within ±30% (100.0%–100.0%), compared with 2.793 (1.711–3.806), 0.926 (0.888–0.964), and 98.1% (94.3%–100.0%) for the PPK model. In this external cohort, the CatBoost–PPK differences excluded zero for V_d_ RMSE and R^2^, but not for all other metrics.

The feature importance scores were calculated based on the CatBoost model to identify the most influential variables for prediction. Higher importance scores indicate a greater impact on the predictive outcome. As shown in Supplementary Table S11, the top three features for predicting CL were weight, eGFR, and sex. The same three variables were also the most important predictors of V_d_. Notably, weight was identified as the most critical feature for both CL and V_d_ predictions, underscoring its significance in predicting vancomycin CL in children.

### Model interpretation

The SHAP analysis was employed to interpret the relationship between variables and the predictive outcomes, including the direction and strength of correlations (Figures 2c,d; Supplementary Figures S4, S5). From Figure 2c, it can be observed that weight and eGFR were positively correlated with CL, while CTS and sex were negatively correlated. Similarly, from Figure 2d, weight and eGFR were positively correlated with V_d_.

Scatter plots and residual plots were analyzed to evaluate the predictive performance of the CatBoost model on the test set for CL and V_d_ (Figures 2e–h; Supplementary Figures S6–S9). In Figures 2e,f, the CatBoost model achieved an R^2^ of 0.89 for CL predictions, with an accuracy of 81.8% within ±30%. In Figures 2g,h, the model achieved an R^2^ of 0.95 for V_d_ predictions, with an accuracy of 92.1% within ±30%. These results collectively demonstrate that the CatBoost model exhibits better predictive performance and good model fit for vancomycin CL in children.

**FIGURE 3:**
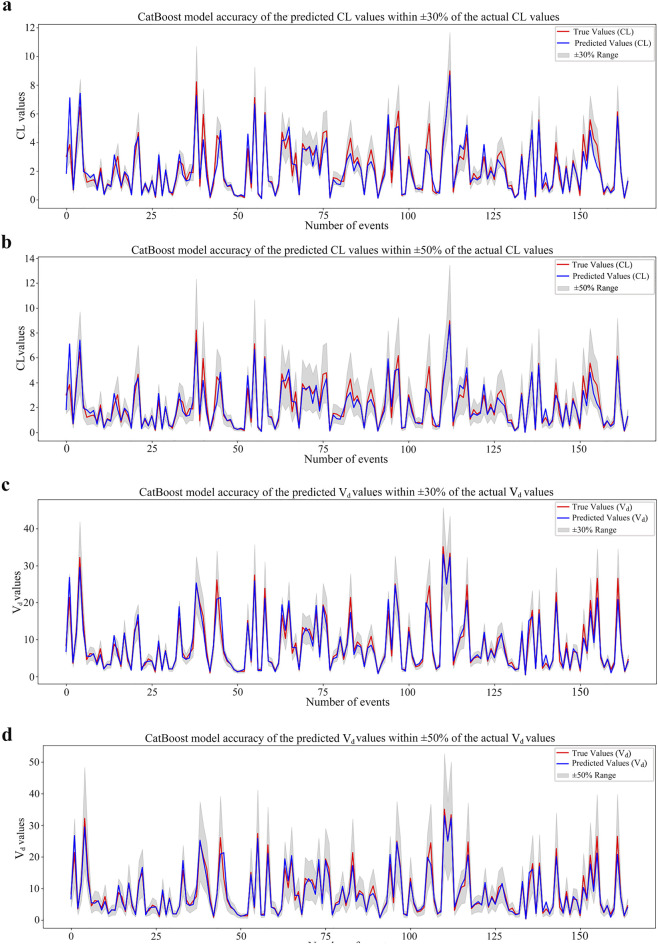
Prediction accuracy of the CatBoost model. **(a)** ±30% prediction accuracy for CL, **(b)** ±50% prediction accuracy for CL, **(c)** ±30% prediction accuracy for V_d_, and **(d)** ±50% prediction accuracy for V._d_.

Time-course plots comparing predicted and observed CL and V_d_ values were generated for the test set (Figure 3). These plots incorporate ±30% and ±50% prediction bounds to assess the model’s clinical applicability. The visualization demonstrates strong temporal concordance between predicted (blue line) and observed values (red line), with the majority of predictions falling within the clinically acceptable ±30% and ±50% range (shaded gray area). This robust tracking performance validates the model’s predictive capability and its potential utility in clinical settings.

### Subgroup analysis based on PMA/age

Subgroup analyses were performed across revised PMA/age and renal-function strata to further assess model robustness. As shown in Tables 4, 5, CatBoost generally showed comparable or better predictive performance than the PPK model across most clinically relevant subgroups.

**TABLE 4:** The validation results of CL and V_d_ in different age subgroups based on the CatBoost/PPK model.

Subgroup	n	RMSE_ML	R^2^_ML	MAE_ML	RMSE_PPK	R^2^_PPK	MAE_PPK
CL (L/h)							
PMA <44 weeks	17	0.07	0.71	0.05	0.08	0.58	0.07
PMA 44 weeks to <1 year	46	0.19	0.79	0.13	0.2	0.76	0.15
1 to <4 years	38	0.51	0.46	0.41	0.55	0.37	0.46
4 to <10 years	38	0.67	0.8	0.54	0.72	0.77	0.6
≥10 years	26	1.07	0.54	0.81	1.22	0.41	0.97
V_d_ (L)							
PMA <44 weeks	17	0.29	0.66	0.21	0.32	0.59	0.26
PMA 44 weeks to <1 year	46	0.49	0.83	0.37	0.6	0.74	0.44
1 to <4 years	38	1.06	0.48	0.85	1.28	0.23	0.97
4 to <10 years	38	2.34	0.86	1.68	2.68	0.81	1.94
≥10 years	26	3.07	0.71	2.59	3.36	0.66	2.74

Age stratification was defined using PMA, for the two youngest groups (PMA <44 weeks and PMA, 44 weeks to <1 year) and chronological age for the older groups (1 to <4 years, 4 to <10 years, and ≥10 years).

**TABLE 5:** Validation results of CL and V_d_ in different renal function subgroups based on the CatBoost/PPK model.

Subgroup	n	RMSE_ML	R^2^_ML	MAE_ML	RMSE_PPK	R^2^_PPK	MAE_PPK
CL (L/h)							
eGFR <60 mL/min	15	0.21	0.96	0.13	0.19	0.97	0.11
eGFR 60 to <90 mL/min	35	0.65	0.80	0.37	0.69	0.77	0.45
eGFR 90 to <120 mL/min	57	0.66	0.83	0.43	0.74	0.79	0.49
eGFR ≥120 mL/min	58	0.56	0.92	0.42	0.63	0.9	0.49
Vd (L)							
eGFR <60 mL/min	15	0.95	0.99	0.60	1.23	0.98	0.73
eGFR 60 to <90 mL/min	35	2.03	0.94	1.18	2.22	0.92	1.4
eGFR 90 to <120 mL/min	57	2.03	0.92	1.27	2.19	0.91	1.29
eGFR ≥120 mL/min	58	1.40	0.96	1.06	1.76	0.93	1.26

Across PMA/age strata, CatBoost maintained stable performance for both CL and V_d_. At the ±30% threshold, CatBoost achieved higher CL prediction accuracy than the PPK model in most age groups, including 4 to <10 years (92.1% vs. 86.8%) and ≥10 years (84.6% vs. 76.9%), while performance was broadly similar in the remaining groups (Supplementary Table S12). For V_d,_ CatBoost also performed favorably, with ±30% accuracies of 89.1%, 92.1%, 94.7%, and 96.2% in the PMA ≥44 weeks to <1 year, 1 to <4 years, 4 to <10 years, and ≥10 years groups, respectively, compared with 87.0%, 84.2%, 92.1%, and 96.2% for the PPK model (Supplementary Table S13). Scatter plots for the age-based subgroup analyses (Supplementary Figures S10–13) showed that most predictions clustered closely around the line of identity and remained largely within the ±30% bounds.

Across renal-function strata, CatBoost likewise showed robust performance. For CL, ±30% accuracy was similar to or higher than that of the PPK model across all eGFR groups, with the clearest advantage observed in patients with eGFR ≥120 mL/min (89.7% vs. 84.5%) (Supplementary Table S14). For V_d_, CatBoost performed similarly or better in all renal subgroups, reaching ±30% accuracies of 91.2% and 96.6% in the eGFR 90 to <120 and ≥120 mL/min groups, respectively, compared with 89.5% and 91.4% for the PPK model (Supplementary Table S15). The corresponding scatter plots (Supplementary Figures S14–S17) further supported good agreement between predicted and observed values across renal-function strata.

## Discussion

This study focused on developing a pre-dose prediction model of vancomycin pharmacokinetic parameters in critically ill children based on ten machine learning and deep learning algorithms. After comparison, CatBoost showed better predictive performance than the other algorithms. Specifically, CatBoost model achieved a RMSE of 0.60, *R* ^2^ of 0.89, MAE of 0.38, and accuracy rate of 81.8% within ±30% error in predicting CL, and a RMSE of 1.75, *R* ^2^ of 0.95, MAE of 1.12, and accuracy rate of 92.1% within ±30% error in predicting V_d_, which are better than those obtained by the PPK model. Bootstrap analysis further supported the robustness of these findings, particularly for CL, although some between-model differences in ±30% accuracy were modest in absolute magnitude. This suggests that the main advantage of the ML–PPK hybrid lies more in improving continuous prediction error and overall robustness than in producing large gains at every categorical accuracy threshold. Taken together, these results indicate that a carefully designed ML–PPK hybrid can outperform a contemporary PPK model when predicting individual PK parameters before any concentration data are available.

From a methodological perspective, it is important to acknowledge that the “true” individual PK parameters were not directly observable in this study. Instead, we approximated them using Bayesian posterior estimates from a previously validated PPK model. This choice reflects current clinical practice, in which multiple vancomycin concentrations (often including both peak and trough) combined with a well-calibrated PPK–Bayesian framework provide the best attainable approximation to patient-specific CL and V_d_. However, for the subgroup of patients with only one concentration measurement, the Bayesian posterior would be expected to shrink more strongly toward the population mean, which may have partially inflated the apparent agreement between the ML model and the PPK-Bayesian targets. Thus, our results show that, given the same baseline covariates, the ML–PPK hybrid approximates the PPK–Bayesian posterior PK parameters more closely than the original parametric covariate model.

TDM guided by Bayesian PPK software is now guideline endorsed and works well once vancomycin concentrations are available (He et al., 2020; Rybak et al., 2020). The remaining bottleneck is designing an individualized initial regimen. Our work directly targets this gap: by predicting CL and V_d_ pre-dose, the ML–PPK hybrid provides a quantitative basis for the first dose, and consistently outperforms the PPK model across most age and renal-function subgroups. With patient-specific CL in hand, the required total daily dose is obtained from the simple equation (Dose = Target AUC_24_/CL). The guideline-recommended AUC_24_ range of 400–600 mgh·L^-1^ for MRSA provides a clinically relevant example; the present framework is not restricted to a single target and can be applied to any exposure goal deemed appropriate for the specific infection and clinical context. This creates a two-step MIPD process. First, the ML model supplies an individualized starting regimen. Second, after TDM, the measured concentrations are analyzed with the PPK-Bayesian tool; if the calculated AUC_24_ falls outside the target window, the regimen is promptly adjusted. Compared with previous approaches, the approach reduces early sub- or supra-therapeutic exposure, potentially improving both efficacy and nephrotoxicity risk. Moreover, the ML model can be embedded in the same clinical decision-support systems that already host PPK software, so bedside complexity does not increase.

Some studies predict vancomycin concentrations or AUC directly (Huang et al., 2021; Chen et al., 2025). Because vancomycin is well described by a one-compartment, first-order model, estimating AUC is essentially the same as estimating CL (Chung et al., 2021). In our work we went one step further: besides CL we also predicted each patient’s V_d_. This brings two benefits. First, V_d_ can signal pathophysiological changes in critically ill children - such as fluid shifts or hypo-albuminemia - that alter drug disposition. Second, with both CL and V_d_, simple pharmacokinetic equations give the full concentration–time curve, allowing clinicians to forecast peak and trough levels in advance. These additional insights make the model easier to use at the bedside.

Compared with PPK modelling, ML algorithms can include more candidate covariates, but adding too many low-value variables quickly makes the model more complex, harder to use at the bedside, and seldom improves accuracy. Seeking a pragmatic balance, we applied an XGBoost-based SFS procedure to the 29 variables available in our dataset. The algorithm retained six high-impact predictors - weight, CTS, eGFR, sex, ICU admission status, and PMA class. Four of these overlap with the covariates identified in our earlier PPK analysis (weight, CTS, eGFR, PMA, albumin), showing that the process identified the main variables in vancomycin pharmacokinetics. Thus, the final ML model uses a covariate set that is similar in size and content to a conventional PPK model, but delivers clearly better predictive performance, striking a favorable balance between accuracy and practicality. We used SHAP analysis to identify the most influential predictors. These plots showed that weight, eGFR, and CTS had the strongest impact on CL, matching the covariate–CL links seen in the PPK model. For volume, SHAP again pointed to weight and eGFR, whereas the PPK model singled out weight and albumin. This small difference likely reflects differences in feature-selection strategy and model structure between the two approaches. Importantly, our ML–PPK hybrid predicts standard PK parameters instead of opaque dose or concentration recommendations. This makes the model behave similarly to a conventional PK model, so clinicians can interpret its outputs using familiar pharmacokinetic reasoning. Taken together, the strong agreement between two independent approaches confirms that these variables are dependable - and clinically meaningful - drivers of vancomycin pharmacokinetics in critically ill children.

Subgroup analyses further showed that the CatBoost model generally maintained performance comparable to or better than the updated PPK model across the revised PMA/age and renal-function strata. Its advantage, however, was not uniform in every subgroup. In some smaller strata, particularly those representing early developmental stages, the performance gap between the two approaches narrowed and occasionally favored the PPK model. This likely reflects a combination of limited subgroup sample size, imbalance in the training distribution, and the difficulty of fully capturing rapid maturation-related changes in vancomycin pharmacokinetics using only pre-dose clinical variables. These issues are especially relevant in younger children, in whom renal maturation, body composition, and hemodynamic instability may change substantially over a short period of time (Padari et al., 2016; Agrawal and Rattan, 2020; Pham, 2020). Notably, performance in the 1 to <4 years subgroup was also only moderate for both models, suggesting that this clinically common age range remains challenging for pre-dose PK prediction. Across renal-function strata, the CatBoost model also remained robust. Its advantage was most apparent in children with higher eGFR, whereas in some lower or intermediate renal-function groups its performance was closer to that of the PPK model. This is clinically plausible, as renal function is a major determinant of vancomycin clearance, but its relationship with drug exposure may become more complex in critically ill children because changes in clearance often coexist with shifts in volume status and other nonrenal factors (Matzke et al., 2011; Economou et al., 2018).

In this study, we applied machine learning techniques to explore the relevant influencing variables for vancomycin modeling from clinical data, automatically extracted EMRs, and monitored physiological data to continuously update the prediction model. Machine learning techniques have demonstrated advantages in dealing with more complex, high-dimensional, and interactive variables from the real world, and have stronger generalization and better accuracy than traditional models (Kruppa et al., 2012; Lee et al., 2018; Mo et al., 2019). CatBoost is a further improved algorithm on the GBDT framework, and it reduces overfitting by regularization, decision tree parameter adjustment, cross validation, and class weight adjustment (Prokhorenkova et al., 2018).

This study also has several limitations. First, this is a retrospective study, which may be influenced by unavoidable confounding factors. Future prospective studies are needed to verify the findings of this study. Second, although an external validation cohort was included, its sample size was relatively small and derived from a single center. Further validation in larger and more diverse external cohorts is still needed. Third, certain dynamic clinical factors were not considered in this study, such as changes in renal function, which could influence vancomycin pharmacokinetic parameters and dosing requirements. To enhance predictive accuracy and clinical applicability, future models should incorporate time-dependent variables. While this study provides useful insights into pre-dose prediction of vancomycin pharmacokinetics in children, further multicenter prospective studies are needed to validate the model in broader populations and to determine whether ML-guided initial dosing can improve early target attainment compared with conventional empirical dosing.

## Conclusion

Optimizing vancomycin therapy in children remains challenging, particularly for the initial regimen in critically ill children. In this multicenter real-world study, we built an ML–PPK hybrid that predicts individual CL and V_d_ before the first dose. The CatBoost model showed robust performance, consistently matching or surpassing the PPK model across clinically relevant subgroups. By providing a simple, interpretable solution to initial dose selection, our approach provides a practical pre-dose extension of vancomycin MIPD. This framework can be integrated into existing clinical decision-support systems to enable patient-specific initial dosing and subsequent TDM-guided adjustment, with the potential to improve the safety and effectiveness of vancomycin therapy in pediatric critical care.

## Data Availability

The raw data supporting the conclusions of this article will be made available by the authors, without undue reservation.
