# Supplementary material for: Machine-learning prediction of pre-dose pharmacokinetics optimizes initial vancomycin dosing in critically ill children
Source: Front Pharmacol. 2026 May 26;17:1839423. doi: 10.3389/fphar.2026.1839423 (PMC13246369; doi:10.3389/fphar.2026.1839423)
Supplement: Supplementary file 1 [file DataSheet1.pdf]

Supplemental information

Supplementary Information. Model development principles 2

Supplementary Tables 8

Supplementary Figures 17

Supplementary Information. Model development principles

1. Feature Token Transformer (FTTransformer)

Feature Token Transformer (FTTransformer), proposed by Yandex in 2021, is designed for classification and regression tasks on structured data[1]. The model encodes categorical and continuous variables separately through embedding layers, combines them, and feeds the resulting representations into self-attention layers for feature interaction. Finally, the predictions are obtained through a linear layer. This approach provides a simpler method for embedding continuous data, thereby improving upon the TabTransformer. By treating features as independent "feature tokens", FTTransformer leverages the self-attention mechanism of the Transformer architecture to capture complex relationships among features. The model dynamically learns feature dependencies and flexibly handles various types of tabular data, including categorical and numerical features, demonstrating superior performance across multiple tasks. In this study, FTTransformer is implemented using PyTorch.

2. Random Forest (RF)

Random Forest (RF) is a powerful machine learning algorithm belonging to the family of ensemble learning methods, first introduced by Leo Breiman in 2001[2]. It performs classification or regression tasks by constructing a large number of decision trees and aggregating their predictions to improve the overall accuracy and robustness of the model. The "random" aspect of Random Forest lies in two key components: first, during the training of each tree, a subset of data points is randomly selected from the original training dataset using bootstrap sampling to create diverse data subsets; second, at each split node within a

tree, only a random subset of features is considered when searching for the optimal split point. In this study, Random Forest is implemented using the Random Forest Classifier.

3. Residual Networks (ResNet)

Residual Networks (ResNet), proposed by the MSRA team led by Kaiming He in 2015, is a type of convolutional neural network (CNN) architecture[3]. By introducing the concept of residual learning, ResNet addresses the issues of gradient vanishing and overfitting in deep neural networks during training, thereby improving the model's performance and trainability. The "residual" aspect of ResNet is reflected in its core structure, where skip connections are employed to directly add the input to the output, forming a residual mapping. This design allows each layer to not only learn the mapping from input to output but also the difference between the input and the target output, significantly enhancing the efficiency of training deep networks. In this study, ResNet is implemented using PyTorch.

4. LinearRegression

LinearRegression is a regression analysis method that models the relationship between one or more independent variables and a dependent variable using a least squares function, known as the linear regression equation[4]. It is used to establish a linear relationship model between the independent and dependent variables. By minimizing the sum of squared residuals (least squares method), it identifies the optimal linear fit to predict the value of the dependent variable. The structure of the linear regression model is simple, easy to understand, and straightforward to implement, making it well-suited for datasets with linear relationships. Additionally, its parameters have clear physical interpretability, allowing for an analysis of the influence of independent variables on the dependent variable. Linear regression can be

categorized into univariate and multivariate types, corresponding to scenarios with a single independent variable and multiple independent variables, respectively. In this study, LinearRegression is implemented using the sklearn library.

5. Multi-Output Support Vector Regression (MSVR)

Multi-Output Support Vector Regression (MSVR) is an extension of Support Vector Regression (SVR) designed to predict multiple continuous target variables simultaneously, making it well-suited for multi-target regression tasks[5]. MSVR combines the advantages of SVR, such as efficiently handling high-dimensional data and strong generalization capabilities, by performing regression through the search for an optimal hyperplane in a high-dimensional feature space. It supports the use of various kernel functions to adapt to different types of data distributions and incorporates regularization parameters to control model complexity, thereby preventing overfitting. MSVR is widely utilized in fields such as financial forecasting, healthcare data analysis, and environmental monitoring, as it effectively captures the relationships among multiple output variables, enabling accurate predictions. In this study, MSVR is implemented using the sklearn library.

6. Extreme Gradient Boosting (XGBoost)

Extreme Gradient Boosting (XGBoost) is an efficient implementation of the Gradient Boosting Decision Tree (GBDT) algorithm, which introduces improvements over traditional GBDT, significantly enhancing model performance[6]. As a forward stagewise additive model, its core lies in employing the ensemble learning framework—specifically the Boosting method—by combining multiple weak learners into a single strong learner. This is achieved through the collective decision-making of multiple trees, where each tree predicts the

difference between the target value and the cumulative predictions of all preceding trees. The final prediction is obtained by summing up the contributions of all trees, thereby improving the overall model performance. XGBoost is composed of multiple Classification and Regression Trees (CART), enabling it to address both classification and regression tasks. In this study, the XGBoost package is used to implement XGBoost.

7. Decision Tree (DT)

Decision Tree (DT), originating in 1966, is a widely used machine learning algorithm that constructs a tree-like structure for classification and regression predictions by recursively partitioning the dataset[7]. In a decision tree, each node represents a feature, each edge corresponds to a splitting condition based on a feature value, and each leaf node denotes a prediction outcome. The core of the algorithm lies in selecting the optimal feature and splitting condition at each node to maximize classification or regression accuracy. Decision trees are not only easy to understand and interpret but also capable of handling various types of data effectively, making them widely applicable in data mining and machine learning tasks. In this study, the Decision Tree Classifier is used to implement the Decision Tree.

8. Category-Boosted Trees (CatBoost)

Category-Boosted Trees (CatBoost) is a gradient boosting decision tree algorithm specifically optimized for categorical features, introduced by Yandex in 2018[8]. It is implemented as a GBDT framework based on symmetric decision trees (oblivious trees) as base learners, featuring fewer parameters, support for categorical variables, and high accuracy. CatBoost primarily addresses the challenge of efficiently and effectively handling categorical features. Additionally, it mitigates issues such as gradient bias and prediction shift, thereby

reducing the risk of overfitting and enhancing both the accuracy and generalization ability of the algorithm. In this study, CatBoost is implemented using the CatBoost library.

9. Artificial neural network (ANN)

An Artificial Neural Network (ANN) is a mathematical or computational model that mimics the structure and functionality of biological neural networks, specifically the central nervous system of animals, particularly the brain[9]. It is used to estimate or approximate functions through computations performed by a large number of interconnected artificial neurons. An ANN consists of three layers: the input layer, the hidden layer(s), and the output layer. The input layer receives the input data, the hidden layer(s) processes the input, and the output layer generates the results. ANNs are capable of solving tasks involving tabular, image, and text data, with the ability to learn weights that map any input to its corresponding output. In this study, ANN is implemented using PyTorch.

10. Light Gradient Boosted Machine (LGBM)

Light Gradient Boosted Machine (LGBM), proposed by Guolin Ke et al. in 2017, provides an efficient and effective implementation of the gradient boosting algorithm[10]. The method introduces two key innovations: Gradient-based One-Side Sampling (GOSS) and Exclusive Feature Bundling (EFB). GOSS is an improvement to the gradient boosting method that focuses on training samples with large gradient magnitudes, thereby accelerating the learning process and reducing computational complexity. EFB, on the other hand, is designed to bundle sparse (mostly zero) and mutually exclusive features, such as one-hot encoded categorical variables. Experimental results on multiple public datasets demonstrate that LGBM can accelerate the training process of traditional GBDT by more than 20 times

while maintaining nearly the same level of accuracy. In this study, the LGBM package is utilized to implement LGBM.

Reference

1. Gorishniy, Y., Rubachev, I., Khrulkov, V. & Babenko, A. Revisiting deep learning models for tabular data. Preprint at http://arxiv.org/abs/2106.11959 (2023).
2. Breiman, L. Random Forests. *Mach. Learn.* **45**, 5–32 (2001).
3. He, K., Zhang, X., Ren, S. & Sun, J. Deep Residual Learning for Image Recognition. Preprint at http://arxiv.org/abs/1512.03385 (2015).
4. Maulud, D. & Abdulazeez, A. M. A Review on Linear Regression Comprehensive in Machine Learning. *J. Appl. Sci. Technol. Trends* **1**, 140–147 (2020).
5. Bao, Y., Xiong, T. & Hu, Z. Multi-step-ahead time series prediction using multiple-output support vector regression. *Neurocomputing* **129**, 482–493 (2014).
6. Chen, T. & Guestrin, C. XGBoost: A scalable tree boosting system. in *Proceedings of the 22nd ACM SIGKDD International Conference on Knowledge Discovery and Data Mining* 785–794 (2016). doi:10.1145/2939672.2939785.
7. Freund, Y. & Schapire, R. E. A decision-theoretic generalization of on-line learning and an application to boosting. *J. Comput. Syst. Sci.* **55**, 119–139 (1997).
8. Prokhorenkova, L., Gusev, G., Vorobev, A., Dorogush, A. V. & Gulin, A. CatBoost: Unbiased boosting with categorical features. Preprint at http://arxiv.org/abs/1706.09516 (2019).
9. Agatonovic-Kustrin, S. & Beresford, R. Basic concepts of artificial neural network (ANN) modeling and its application in pharmaceutical research. *J. Pharm. Biomed. Anal.* **22**, 717–727 (2000).
10. Ke, G. *et al.* LightGBM: A highly efficient gradient boosting decision tree. (2017)

Supplementary Tables

Table S1. Ten-fold cross-validation candidate parameters

Algorithm	Parameter
FTTransformer	param_grid = {<br>'dim': [16, 32, 64, 128],<br>'depth': [3, 4, 5, 6, 7, 8],<br>'heads': [2, 4, 6, 8],<br>}
RF	param_grids = {<br>'n_estimators': [50, 100, 150, 200, 250, 300],<br>'max_depth': [4, 5, 6, 7],<br>'min_samples_split': [2, 5, 10],<br>'min_samples_leaf': [1, 2, 4, 10],<br>'max_features': ['auto', 'sqrt'],<br>}
ResNet	param_grids = {<br>'hidden_dims': [[64, 128, 256, 512], [16, 32, 64, 128], [32, 64, 128, 256]],<br>'activation': ['relu', 'selu', 'elu', 'leaky_relu'],<br>}
LinearRegression	param_grid = {<br>'fit_intercept': [True, False],<br>'normalize': [True, False],<br>}
MSVR	param_grid = {<br>'kernel': ['linear', 'poly', 'rbf', 'sigmoid'],<br>'C': [0.001, 0.003, 0.01, 0.1, 1.0],<br>'epsilon': [0.1, 0.3, 0.5, 0.7, 1.0],<br>'coef0': [-1, 0, 1],<br>'degree': [3, 4, 5, 6, 7, 8],<br>}
XGBoost	param_grids = {<br>'n_estimators': [50, 100, 150, 200, 250, 300],<br>'learning_rate': [0.01, 0.015, 0.1, 0.008],<br>'max_depth': [4, 5, 6],<br>}
DT	param_grid = {<br>'max_depth': [3, 4, 5, 6, 7, 8, None],<br>'max_features': [None, 'log2', 'sqrt']<br>}
CatBoost	param_grids = {<br>'learning_rate': [0.005, 0.008, 0.01, 0.03, 0.05, 0.1, 0.3],

	'depth': [3, 4, 5, 6],<br>'subsample': [0.8, 0.9, 1.0],<br>'rsm': [0.8, 0.9, 1.0],<br>}
ANN	param_grids = {<br>'hidden_dim': [16, 32, 64, 128, 256],<br>'activation': ['relu', 'selu', 'elu', 'leaky_relu', 'swish']<br>}
LGBM	param_grids = {<br>'n_estimators': [50, 100, 150, 200, 250, 300],<br>'max_depth': [3, 4, 5, 6],<br>'learning_rate': [0.001, 0.003, 0.005, 0.01, 0.03],<br>}

Abbreviations: FTTransformer, feature token transformer; RF, random forest; ResNet, residual networks; MSVR, multi-output support vector regression; XGBoost, extreme gradient boosting; DT, decision tree; CatBoost, category-boosted trees; ANN, artificial neural network; LGBM, light gradient boosted machine. .

Table S2. The optimal parameters of ten models

Algorithm	Parameter
FTTransformer	dim=16, depth=6, heads=8
RF	n_estimators=200, max_depth=5, min_samples_split=10, min_samples_leaf=4, max_features=sqrt
ResNet	hidden_dims=[32, 64, 128, 256], activation=leaky_relu
LinearRegression	fit_intercept=True, normalize=False
MSVR	C=0.003, coef0=1, degree=3, epsilon=0.01, kernel=poly
XGBoost	n_estimators=100, learning_rate=0.015, max_depth=4
DT	max_depth=6, max_features=log2
CatBoost	learning_rate=0.008, depth=3, subsample=0.8, rsm=1.0
ANN	hidden_dim=256, activation=swish
LightGBM	n_estimators=50, max_depth=5, learning_rate=0.3

Abbreviations: FTTransformer, feature token transformer; RF, random forest; ResNet, residual networks; MSVR, multi-output support vector regression; XGBoost, extreme

gradient boosting; DT, decision tree; CatBoost, category-boosted trees; ANN, artificial neural network; LGBM, light gradient boosted machine.

Table S3. Baseline characteristics of the study cohorts by center

Variable	Xinhua Hospital	People's Hospital of the Inner Mongolia Autonomous Region	Shanghai Children's Medical Center (external cohort)
Patients, n	753	68	53
Age (years), median (IQR)	1.80 (0.51–6.45)	0.64 (0.10–6.04)	6.69 (3.82–13.06)
PMA (weeks), median (IQR)	134.0 (65.9–376.4)	73.4 (40.5–354.8)	389 (239–721)
Height (cm), median (IQR)	83.0 (64.0–117.7)	66.0 (40.0–110.3)	125 (98–160)
Weight (kg), median (IQR)	11.5 (6.5–20.0)	8.0 (2.6–19.3)	20.0 (16.0–50.0)
Male sex, n (%)	435 (57.8)	43 (63.2)	36 (67.9)
ICU admission, n (%)	624 (82.9)	51 (75.0)	53 (100)
Cardiothoracic surgery, n (%)	85 (11.3)	1 (1.5)	0 (0)
Serum creatinine (μmol/L), median (IQR)	23.6 (17.3–32.8)	26.9 (19.0–39.4)	37.0 (19.0–57.0)
Blood urea nitrogen (mmol/L), median (IQR)	3.6 (2.4–5.0)	4.2 (2.9–5.8)	6.8 (5.1–10.2)
eGFR (mL/min/1.73 m^2), median (IQR)	109.3 (86.6–140.5)	88.7 (59.2–115.2)	82.2 (54.9–125.4)
Albumin (g/L), median (IQR)	36.6 (31.8–40.6)	32.8 (30.4–38.4)	36.7 (33.7–42.2)

Abbreviations: PMA, postmenstrual age; ICU, intensive care unit; eGFR, estimated glomerular filtration rate.

Table S4. Comparison of parameters between the original model and the updated model

Parameter	Original model		Updated model	
	Value	RSE (%)	Value	RSE (%)
θ_{CL}	7.75	2.3	7.84	1.9
TM_{50}	37.0	4.6	37.1	3.8
Hill	-1.63	13	-1.64	9.1
θ_{eGFR}	1.01	3.4	1.02	3.3
θ_1	0.761	3.6	0.783	3.2
θ_V	36.2	1.7	36.6	1.6
θ_{ALB}	0.279	26.7	0.255	25.9
CL (%CV)	27.5	6.0	28.3	5.8
V_d (%CV)	27.2	16.2	27.2	16.6
Cov (CL - V_d)	0.0536	10.8	0.0548	7.6
Exponential (%CV)	17.7	10.6	17.7	10.7
Additive (mg/L)	0.154	44.5	0.154	37.7

Notes: The calculation formulas are as follows:

$$CL=\theta_{CL}\times\left(\frac{WT}{70}\right)^{0.75}\times\left[\frac{1}{1+\left(\frac{PMA}{TM_{50}}\right)^{Hill}}\right]\times\left(\frac{eGFR}{109}\right)^{\theta_{eGFR}}\times\theta_1^{CTS}$$

$$V_d=\theta_V\times\frac{WT}{70}\times\left(\frac{36.2}{ALB}\right)^{\theta_{ALB}}$$

$$eGFR = 40.7\times\left(\frac{\text{height (m)}}{Scr}\right)^{0.64}\times\left(\frac{30}{BUN}\right)^{0.202}$$

Abbreviations: CTS, cardiothoracic surgery (CTS patients: CTS = 1; non-CTS patients: CTS = 0); CL, clearance (L/h); V_d, volume of distribution (L); WT, total body weight (kg); ALB, albumin (g/L); BUN: blood urea nitrogen (mg/dL); SCr, serum creatinine (mg/dL); PMA, postmenstrual age (weeks); eGFR, estimated glomerular filtration rate (mL/min/1.73m^2); TM_{50}: value of PMA when 50% of maturation of CL has been reached and Hill: slope of sigmoid model described in the maturation function; RSE, relative standard error; %CV, coefficient of variation; Cov, Covariance.

Table S5. The results of ten-fold cross-validation for CL (L/h)

Algorithm	RMSE	R^2	MAE
FTTransformer	1.03 ± 0.25	0.71 ± 0.06	0.66 ± 0.11
RF	0.89 ± 0.30	0.79 ± 0.06	0.60 ± 0.11
ResNet	0.86 ± 0.18	0.80 ± 0.05	0.57 ± 0.08
LinearRegression	1.03 ± 0.27	0.72 ± 0.05	0.75 ± 0.12
MSVR	0.85 ± 0.23	0.81 ± 0.05	0.57 ± 0.11
XGBoost	0.90 ± 0.30	0.78 ± 0.06	0.62 ± 0.12
DT	0.93 ± 0.23	0.77 ± 0.05	0.60 ± 0.09
CatBoost	0.79 ± 0.20	0.82 ± 0.07	0.49 ± 0.08
ANN	0.90 ± 0.23	0.78 ± 0.06	0.63 ± 0.13
LightGBM	0.90 ± 0.29	0.78 ± 0.06	0.62 ± 0.11

Abbreviations: FTTransformer, feature token transformer; RF, random forest; ResNet, residual networks; MSVR, multi-output support vector regression; XGBoost, extreme gradient boosting; DT, decision tree; CatBoost, category-boosted trees; ANN, artificial neural network; LGBM, light gradient boosted machine; R^2, coefficient of determination; RMSE, root mean square error; MAE, mean absolute error.

Table S6. The results of ten-fold cross-validation for V_d (L)

Algorithm	RMSE	R^2	MAE
FTTransformer	3.79 ± 1.31	0.76 ± 0.09	1.91 ± 0.43
RF	3.05 ± 1.03	0.84 ± 0.06	2.00 ± 0.30
ResNet	2.65 ± 0.59	0.87 ± 0.05	1.65 ± 0.22
LinearRegression	3.69 ± 1.01	0.76 ± 0.07	2.63 ± 0.43
MSVR	2.55 ± 0.81	0.89 ± 0.04	1.67 ± 0.30
XGBoost	2.88 ± 0.99	0.86 ± 0.05	1.87 ± 0.30
DT	2.98 ± 1.21	0.85 ± 0.07	1.74 ± 0.46
CatBoost	2.32 ± 0.49	0.90 ± 0.03	1.39 ± 0.16
ANN	2.46 ± 0.77	0.89 ± 0.04	1.62 ± 0.32
LightGBM	2.80 ± 1.06	0.86 ± 0.06	1.83 ± 0.31

Abbreviations: FTTransformer, feature token transformer; RF, random forest; ResNet, residual networks; MSVR, multi-output support vector regression; XGBoost, extreme

gradient boosting; DT, decision tree; CatBoost, category-boosted trees; ANN, artificial neural network; LGBM, light gradient boosted machine; R^2, coefficient of determination; RMSE, root mean square error; MAE, mean absolute error.

Table S7. Bootstrap 95% confidence intervals for model performance in test set

Parameter	Metric	CatBoost	PPK	Δ (CatBoost − PPK)
CL (L/h)	RMSE	0.596 (0.471, 0.725)	0.659 (0.534, 0.796)	-0.063 (-0.116, -0.011)
	R^2	0.891 (0.828, 0.933)	0.867 (0.794, 0.914)	0.024 (0.004, 0.047)
	MAE	0.385 (0.319, 0.456)	0.445 (0.375, 0.523)	-0.060 (-0.100, -0.022)
	±30% accuracy	81.8% (75.8%, 87.3%)	77.0% (70.3%, 83.0%)	4.8% (-0.6%, 10.3%)
V_d (L)	RMSE	1.753 (1.407, 2.096)	1.982 (1.565, 2.411)	-0.230 (-0.451, 0.005)
	R^2	0.946 (0.921, 0.965)	0.931 (0.891, 0.958)	0.015 (-0.000, 0.036)
	MAE	1.115 (0.924, 1.325)	1.250 (1.029, 1.486)	-0.135 (-0.269, -0.001)
	±30% accuracy	92.1% (87.9%, 96.4%)	89.7% (84.8%, 93.9%)	2.4% (-1.8%, 6.7%)

Values are presented as estimate (bootstrap 95% CI) based on resampling with replacement (5,000 replicates). Abbreviations: CL, clearance; V_d, volume of distribution; RMSE, root mean square error; MAE, mean absolute error; R^2, coefficient of determination.

Table S8. The results of external validation cohort

Algorithm	CL (L/h)			V_d (L)		
	RMSE	R^2	MAE	RMSE	R^2	MAE
CatBoost	0.71	0.85	0.39	2.38	0.95	1.33
PPK	0.79	0.81	0.50	2.80	0.93	1.63

Abbreviations: CatBoost, category-boosted trees; PPK, population pharmacokinetics; R^2, coefficient of determination; RMSE, root mean square error; MAE, mean absolute error.

Table S9. Accuracy (%) of CL and V_d on external validation cohort

Algorithm	CL (L/h)			V_d (L)		
	±20%	±30%	±50%	±20%	±30%	±50%
CatBoost	69.8	84.9	96.2	90.6	100.0	100.0
PPK	66.0	79.2	96.2	88.7	98.1	100.0

Abbreviations: CatBoost, category-boosted trees; PPK, population pharmacokinetics.

Table S10. Bootstrap 95% confidence intervals for model performance in the external validation cohort

Parameter	Metric	CatBoost	PPK	Δ (CatBoost − PPK)
CL (L/h)	RMSE	0.713 (0.373, 1.009)	0.793 (0.449, 1.110)	-0.080 (-0.199, 0.046)
	R^2	0.847 (0.644, 0.951)	0.810 (0.593, 0.929)	0.037 (-0.024, 0.095)
	MAE	0.387 (0.242, 0.567)	0.498 (0.352, 0.683)	-0.112 (-0.190, -0.033)
	±30% accuracy	84.9% (75.4%, 94.3%)	79.2% (67.9%, 88.7%)	5.7% (-5.7%, 17.0%)
V_d (L)	RMSE	2.378 (1.324, 3.382)	2.793 (1.711, 3.806)	-0.415 (-0.818, -0.070)
	R^2	0.947 (0.915, 0.979)	0.926 (0.888, 0.964)	0.020 (0.003, 0.042)
	MAE	1.330 (0.862, 1.931)	1.630 (1.088, 2.293)	-0.300 (-0.648, 0.005)
	±30% accuracy	100.0% (100.0%, 100.0%)	98.1% (94.3%, 100.0%)	1.9% (0.0%, 5.7%)

Values are presented as estimate (bootstrap 95% CI) based on resampling with replacement (5,000 replicates). Abbreviations: CL, clearance; V_d, volume of distribution; RMSE, root mean square error; MAE, mean absolute error; R^2, coefficient of determination.

Table S11. Variable importance scores based on the CatBoost model (CL/V_d)

Feature	Importance_CL	Importance_V_d
Weight	78.15	94.68
eGFR	18.49	3.22
Sex	1.24	0.86
CTS	1.05	0.50
ICU	0.80	0.44
PMA class	0.31	0.29

Abbreviations: eGFR, estimated glomerular filtration rate; CTS, after cardiac surgery; ICU, intensive care unit; PMA, postmenstrual age.

Table S12. The accuracy (%) of CL in different age subgroups based on the CatBoost/PPK model

Subgroup	CatBoost			PPK		
	±20%	±30%	±50%	±20%	±30%	±50%
PMA <44 weeks	52.9	70.6	88.2	47.1	52.9	82.4
PMA 44 weeks to <1 year	69.6	80.4	89.1	58.7	82.6	91.3
1 to <4 years	47.4	76.3	89.5	42.1	71.1	86.8
4 to <10 years	57.9	92.1	100	60.5	86.8	97.4
≥10 years	69.2	84.6	92.3	61.5	76.9	92.3

Table S13. The accuracy (%) of V_d in different age subgroups based on the CatBoost/PPK model

Subgroup	CatBoost			PPK		
	±20%	±30%	±50%	±20%	±30%	±50%
PMA <44 weeks	76.5	88.2	100	76.5	94.1	94.1
PMA 44 weeks to <1 year	76.1	89.1	100	76.1	87.0	97.8
1 to <4 years	81.6	92.1	94.7	73.7	84.2	94.7
4 to <10 years	78.9	94.7	97.4	76.3	92.1	97.4
≥10 years	88.5	96.2	100	76.9	96.2	96.2

Table S14. The accuracy (%) of CL in different renal function subgroups based on the CatBoost/PPK model

Subgroup	CatBoost			PPK		
	±20%	±30%	±50%	±20%	±30%	±50%
eGFR <60 ml/min	53.3	60.0	73.3	40.0	60.0	86.7
eGFR 60 to <90 ml/min	51.4	77.1	91.4	37.1	65.7	85.7
eGFR 90 to <120 ml/min	57.9	82.5	93.0	57.9	80.7	91.2
eGFR ≥120 ml/min	69.0	89.7	96.6	65.5	84.5	94.8

Table S15. The accuracy (%) of V_d in different renal function subgroups based on the CatBoost/PPK model

Subgroup	CatBoost			PPK		
	±20%	±30%	±50%	±20%	±30%	±50%
eGFR <60 ml/min	80.0	86.7	100.0	80.0	86.7	93.3
eGFR 60 to <90 ml/min	80.0	88.6	100.0	68.6	88.6	94.3
eGFR 90 to <120 ml/min	77.2	91.2	98.3	71.9	89.5	98.2
eGFR ≥120 ml/min	82.8	96.6	96.6	82.8	91.4	96.6

Supplementary Figures

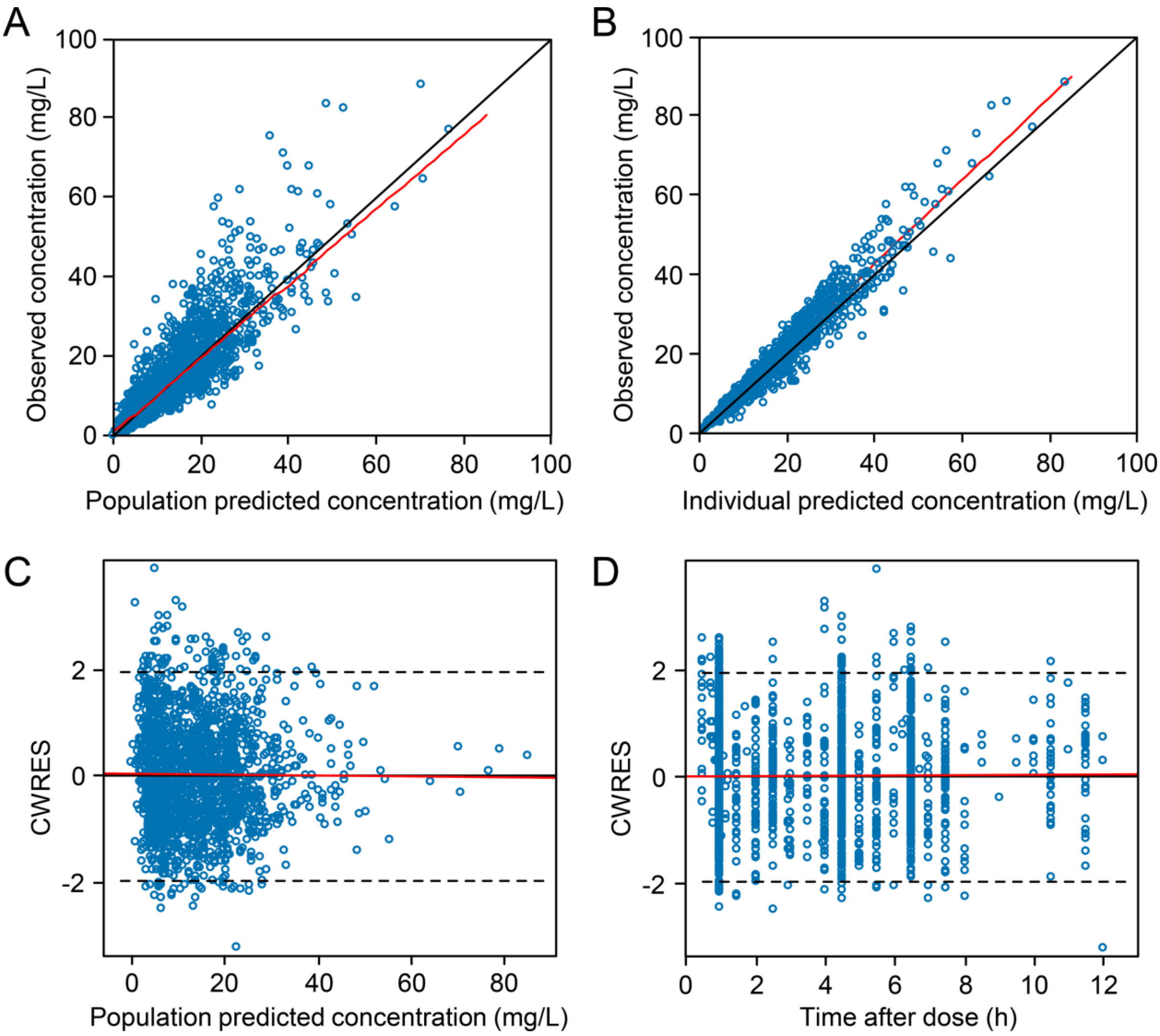

Figure S1. Goodness-of-fit diagnostic plots of the updated model. (A) Observation (DV) vs. population prediction (PRED). (B) DV vs. individual prediction (IPRED). (C) conditional weighted residual errors (CWRES) vs. PRED. (D) CWRES vs. time after dose. The black solid lines are the reference lines, and red solid lines are the loess smooth lines.

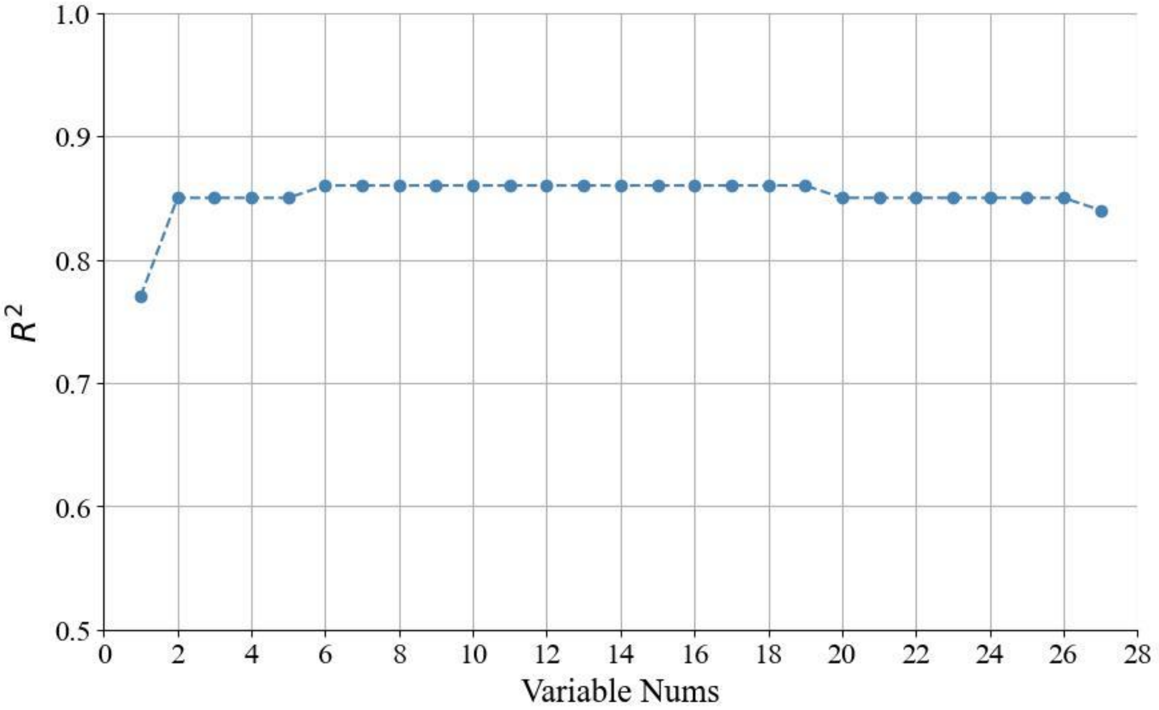

Figure S2. Stepwise forward feature selection results.

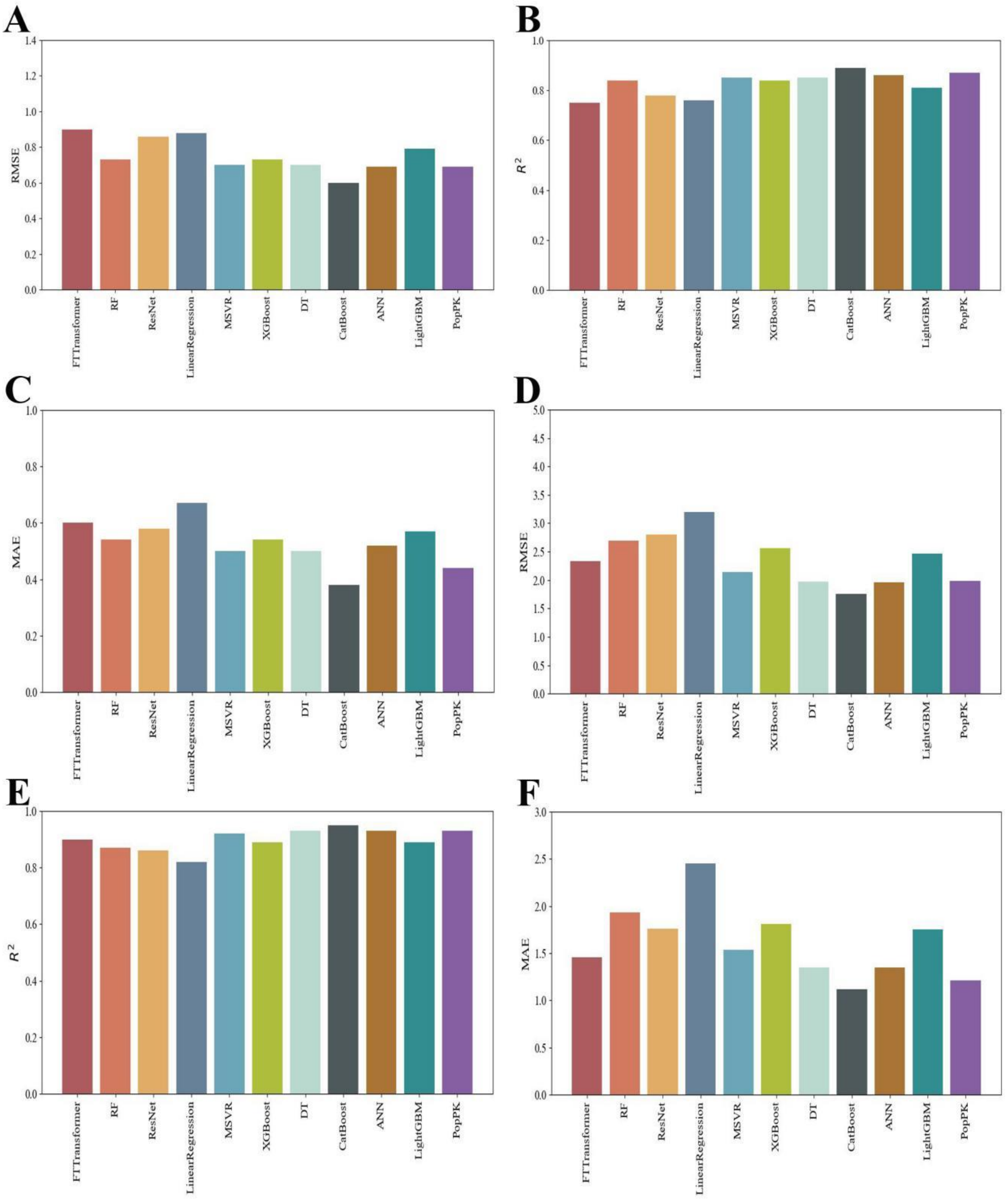

Figure S3. Evaluation metrics of the test set based on the CatBoost model. A) show RMSE (mean ± std) of the training set and test set based on CL was calculated; B) show R^2 (mean ± std) of the training set and test set based on CL was calculated; C) show MAE (mean ± std) of the training set and test set based on CL was calculated; D) show RMSE (mean ± std) of the training set and test set based on V_d was calculated; E) show R^2 (mean ± std) of the

training set and test set based on V_d was calculated; F) show MAE (mean ± std) of the training set and test set based on V_d was calculated.

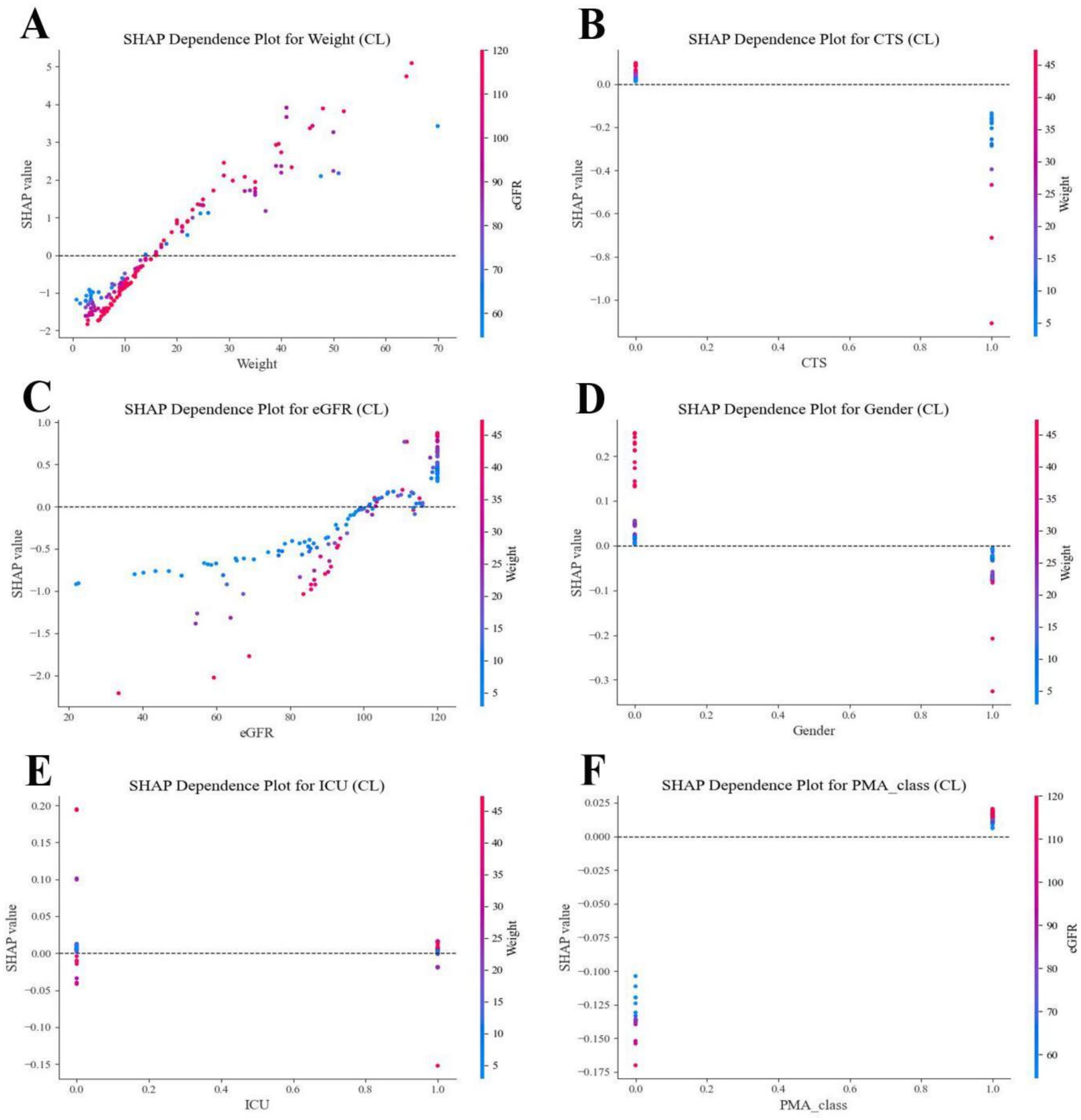

Figure S4. SHAP dependency diagram based on CatBoost model for CL. Dot colors become redder as the feature value increases and bluer as the feature value decreases. SHAP value indicates the impact of each variable on the model's output. A) weight, B) CTS, C) eGFR, D) sex, E) ICU, F) PMA_class.

Abbreviations: CTS, after cardiac surgery; ICU, intensive care unit; eGFR, estimated glomerular filtration rate; PMA, postmenstrual age.

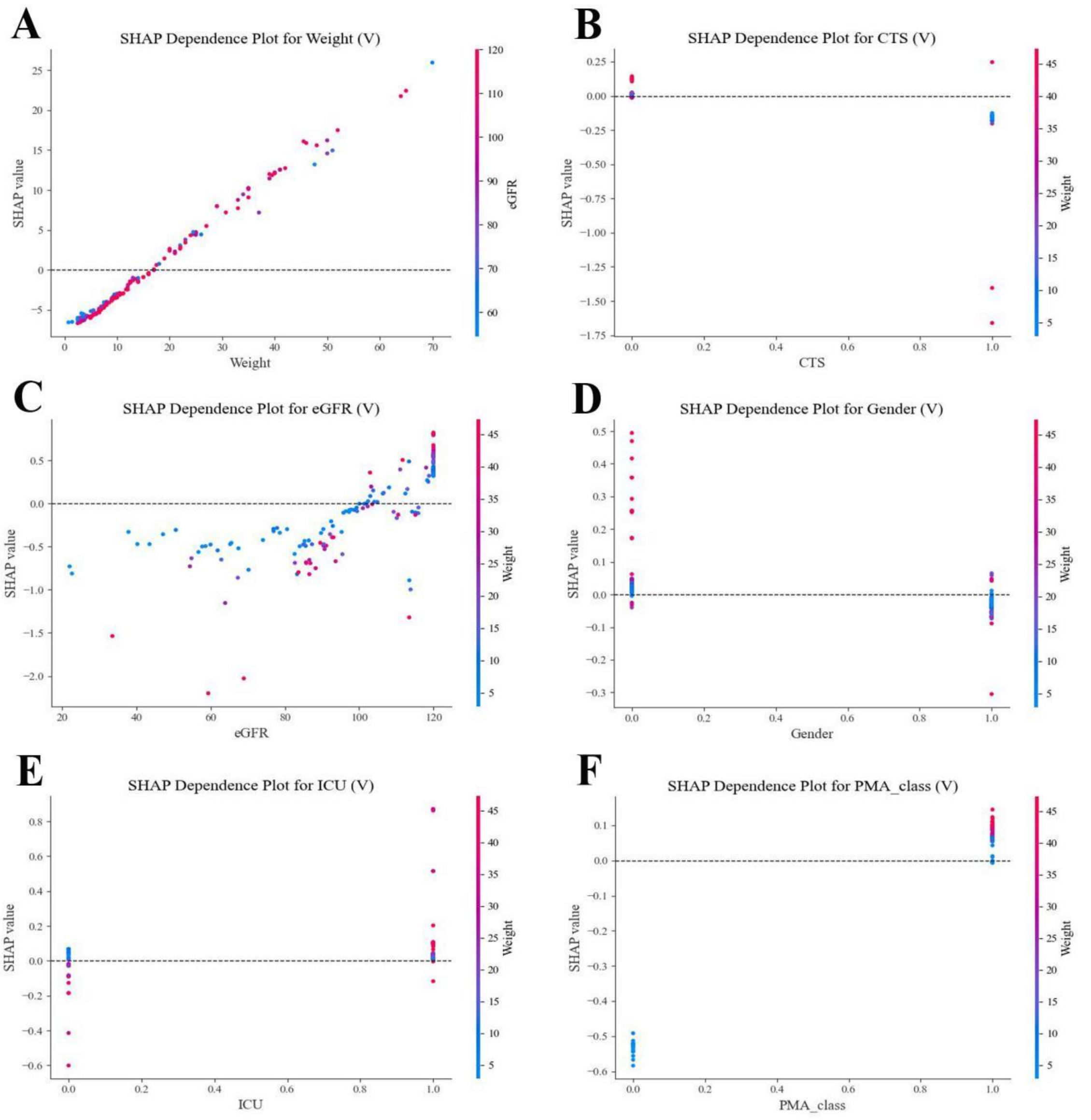

Figure S5. SHAP dependency diagram based on CatBoost model for V_d. Dot colors become redder as the feature value increases and bluer as the feature value decreases. SHAP value indicates the impact of each variable on the model's output. A) weight, B) CTS, C) eGFR, D) sex, E) ICU, and F) PMA_class.

Abbreviations: CTS, after cardiac surgery; ICU, intensive care unit; eGFR, estimated glomerular filtration rate; PMA, postmenstrual age.

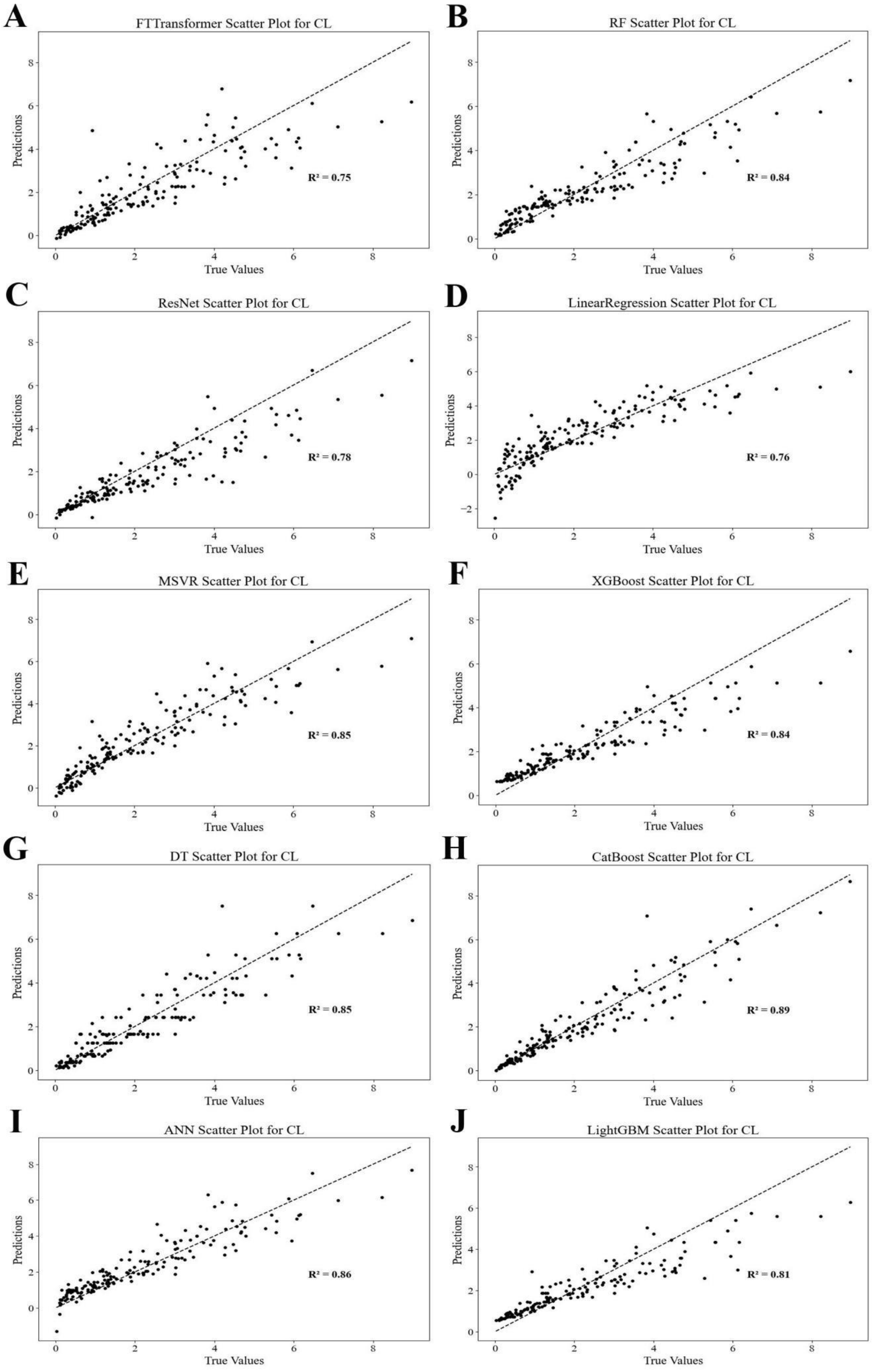

Figure S6. Scatter plots of ten models for CL. A) FTTransformer, B) RF, C) ResNet, D) LinearRegression, E) MSVR, F) XGBoost, G) DT, H) CatBoost, I) ANN, and J) LightGBM.

FTTransformer, feature token transformer; RF, random forest; ResNet, residual networks; MSVR, multi-output support vector regression; XGBoost, extreme gradient boosting; DT, decision tree; CatBoost, category-boosted trees; ANN, artificial neural network; LGBM, light gradient boosted machine; R^2, coefficient of determination.

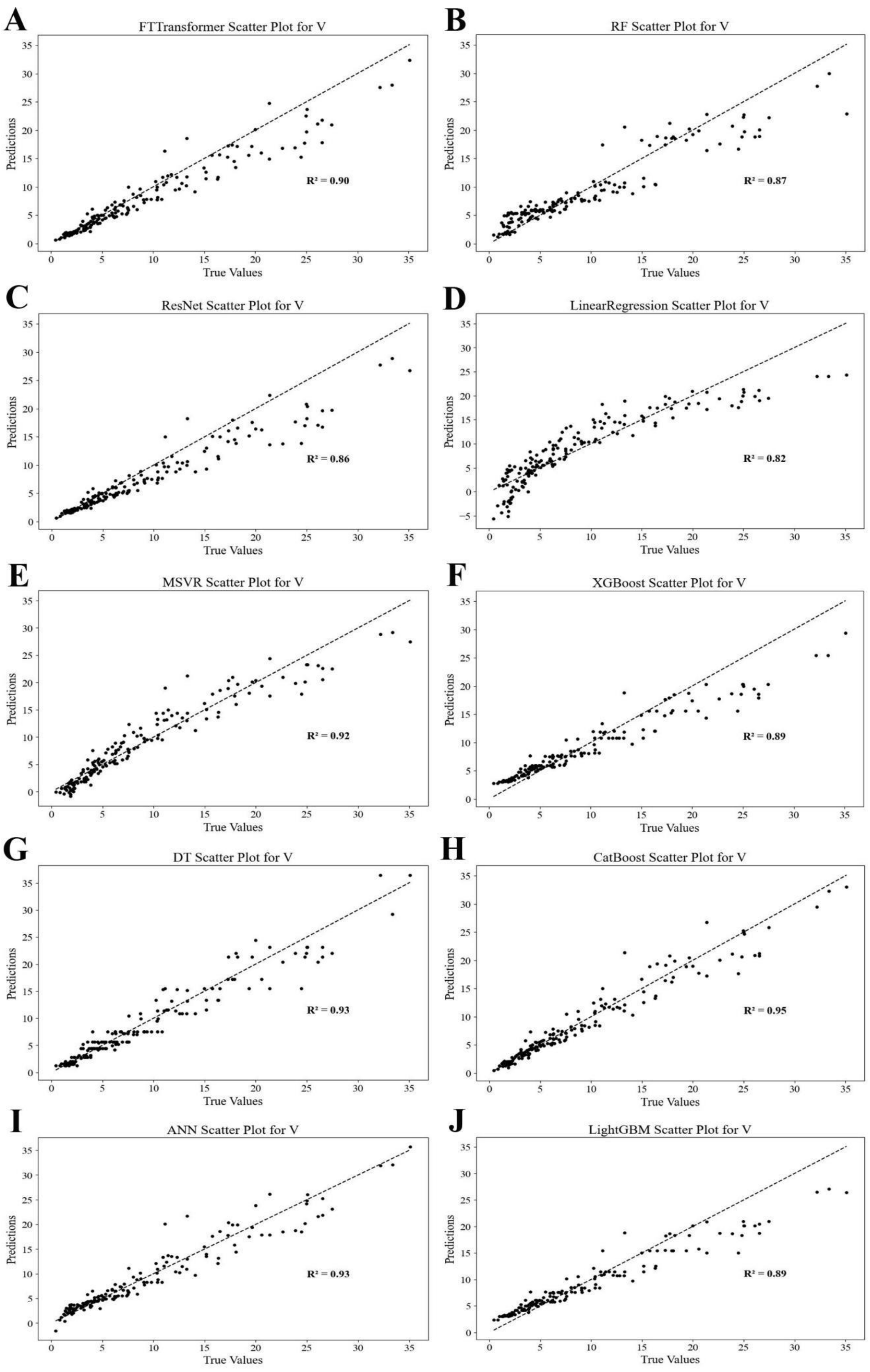

Figure S7. Scatter plots of ten models for V_d. A) FTTransformer, B) RF, C) ResNet, D) LinearRegression, E) MSVR, F) XGBoost, G) DT, H) CatBoost, I) ANN, and J) LightGBM.

FTTransformer, feature token transformer; RF, random forest; ResNet, residual networks; MSVR, multi-output support vector regression; XGBoost, extreme gradient boosting; DT, decision tree; CatBoost, category-boosted trees; ANN, artificial neural network; LGBM, light gradient boosted machine; R^2, coefficient of determination.

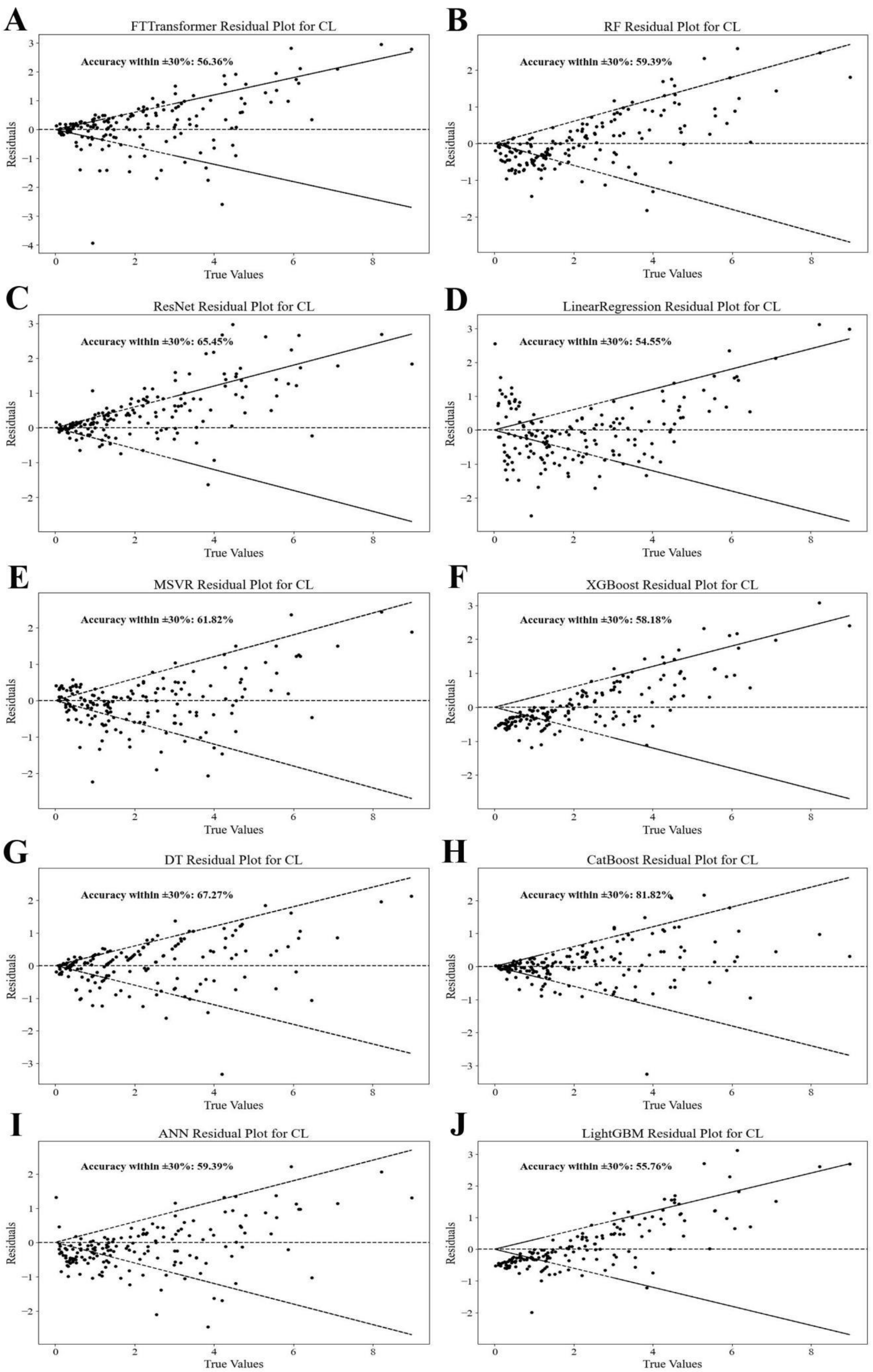

Figure S8. Residual plots of ten models for CL. A) FTTransformer, B) RF, C) ResNet, D) LinearRegression, E) MSVR, F) XGBoost, G) DT, H) CatBoost, I) ANN, and J) LightGBM.

FTTransformer, feature token transformer; RF, random forest; ResNet, residual networks; MSVR, multi-output support vector regression; XGBoost, extreme gradient boosting; DT, decision tree; CatBoost, category-boosted trees; ANN, artificial neural network; LGBM, light gradient boosted machine; R^2, coefficient of determination.

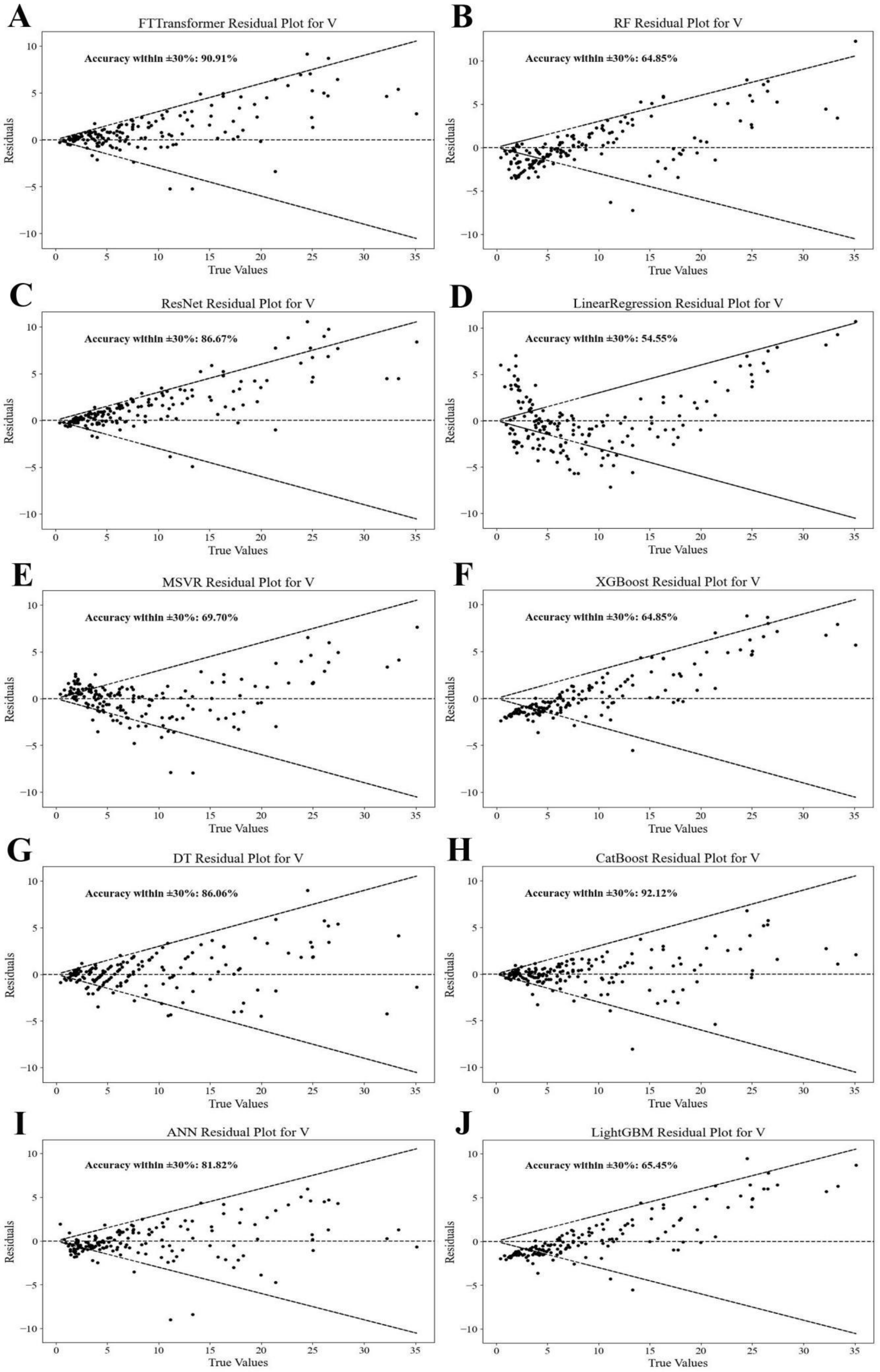

Figure S9. Residual plots of ten models for V_d. A) FTTransformer, B) RF, C) ResNet, D) LinearRegression, E) MSVR, F) XGBoost, G) DT, H) CatBoost, I) ANN, and J) LightGBM.

FTTransformer, feature token transformer; RF, random forest; ResNet, residual networks; MSVR, multi-output support vector regression; XGBoost, extreme gradient boosting; DT, decision tree; CatBoost, category-boosted trees; ANN, artificial neural network; LGBM, light gradient boosted machine; R^2, coefficient of determination.

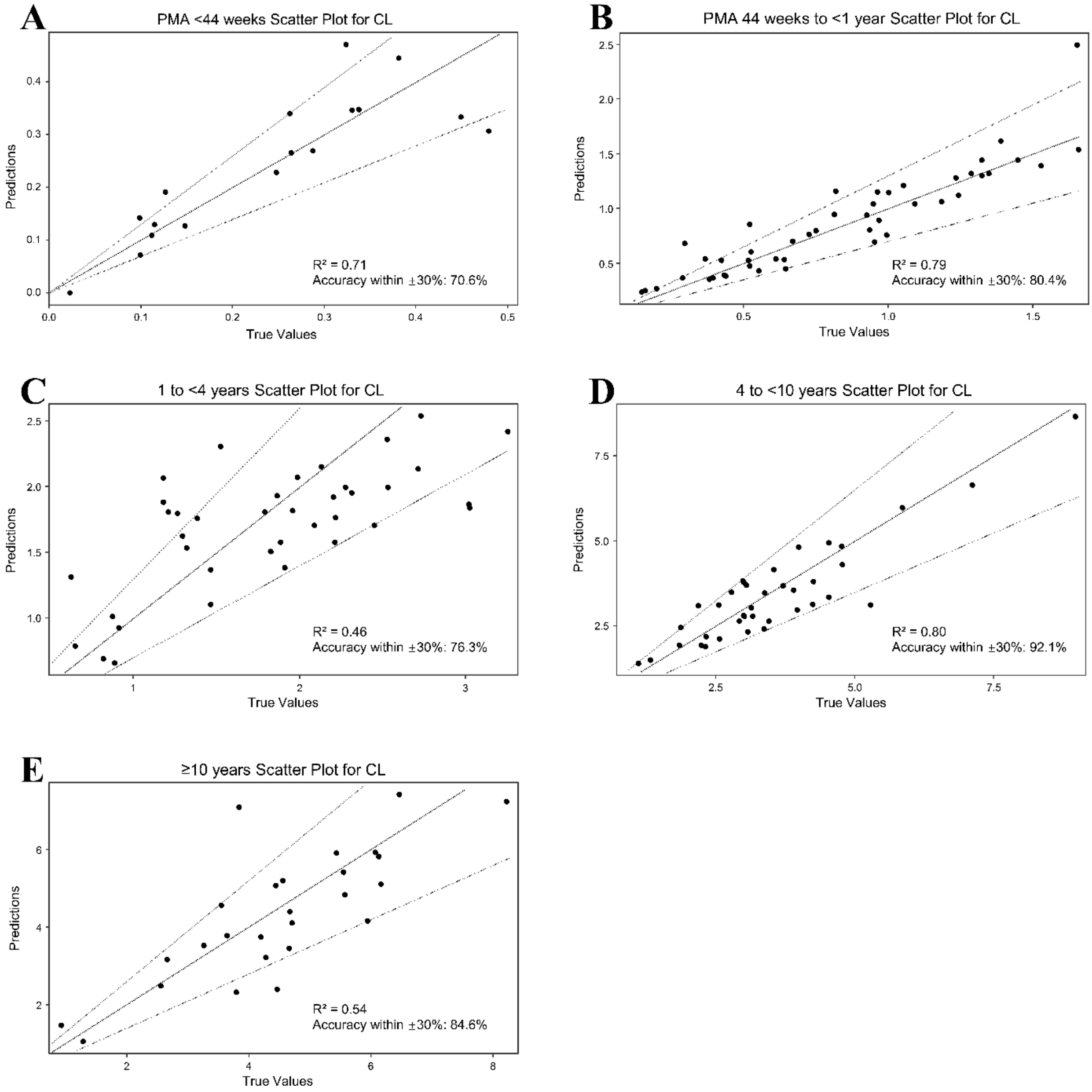

Figure S10. Scatter plots of CL for different age subgroups based on the CatBoost model. A) PMA <44 weeks, B) PMA ≥44 weeks to <1 year, C) 1 to <4 years, D) 4 to <10 years, and E) ≥10 years.

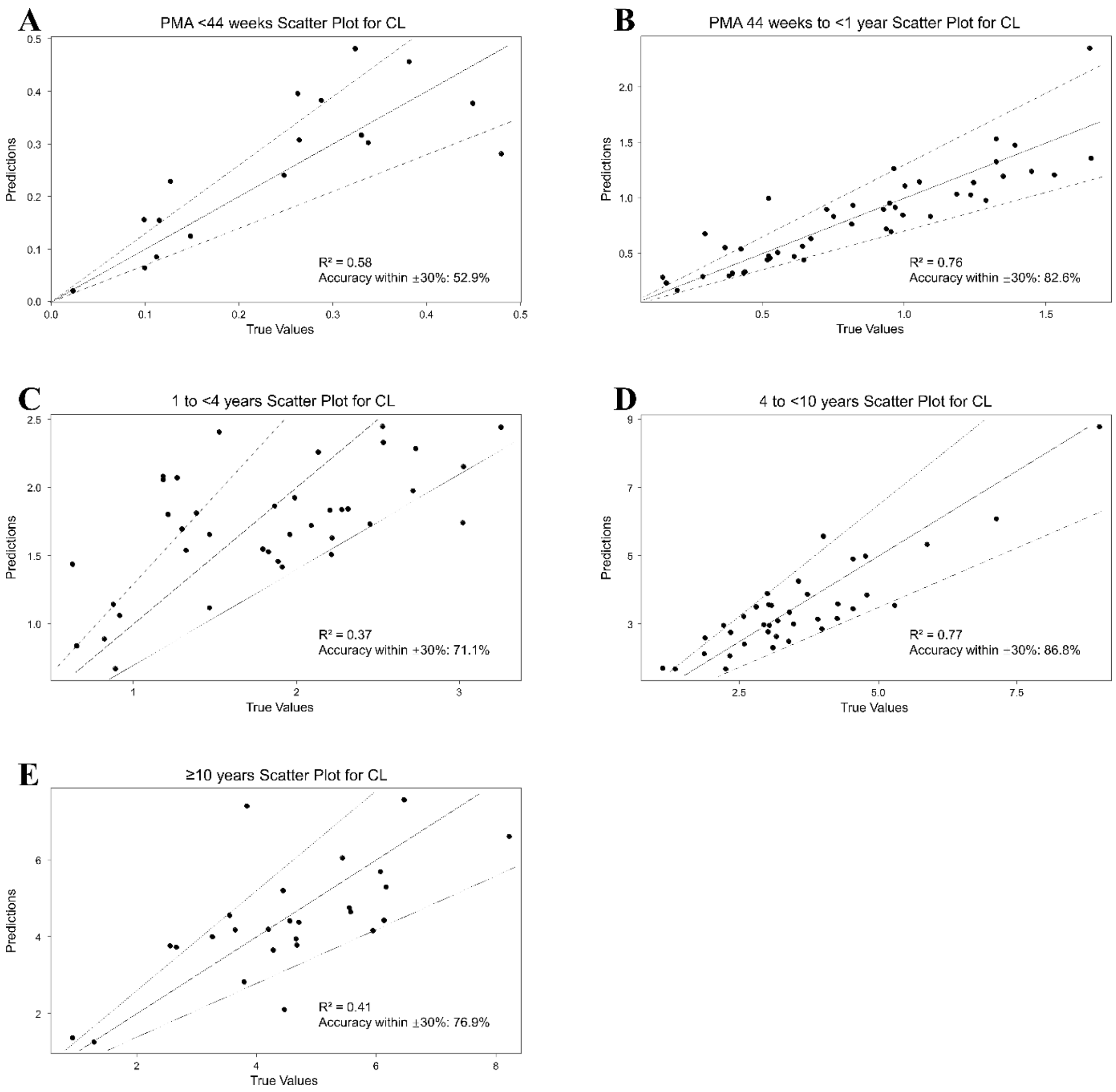

Figure S11. Scatter plots of CL for different age subgroups based on the PPK model. A) PMA <44 weeks, B) PMA ≥44 weeks to <1 year, C) 1 to <4 years, D) 4 to <10 years, and E) ≥10 years.

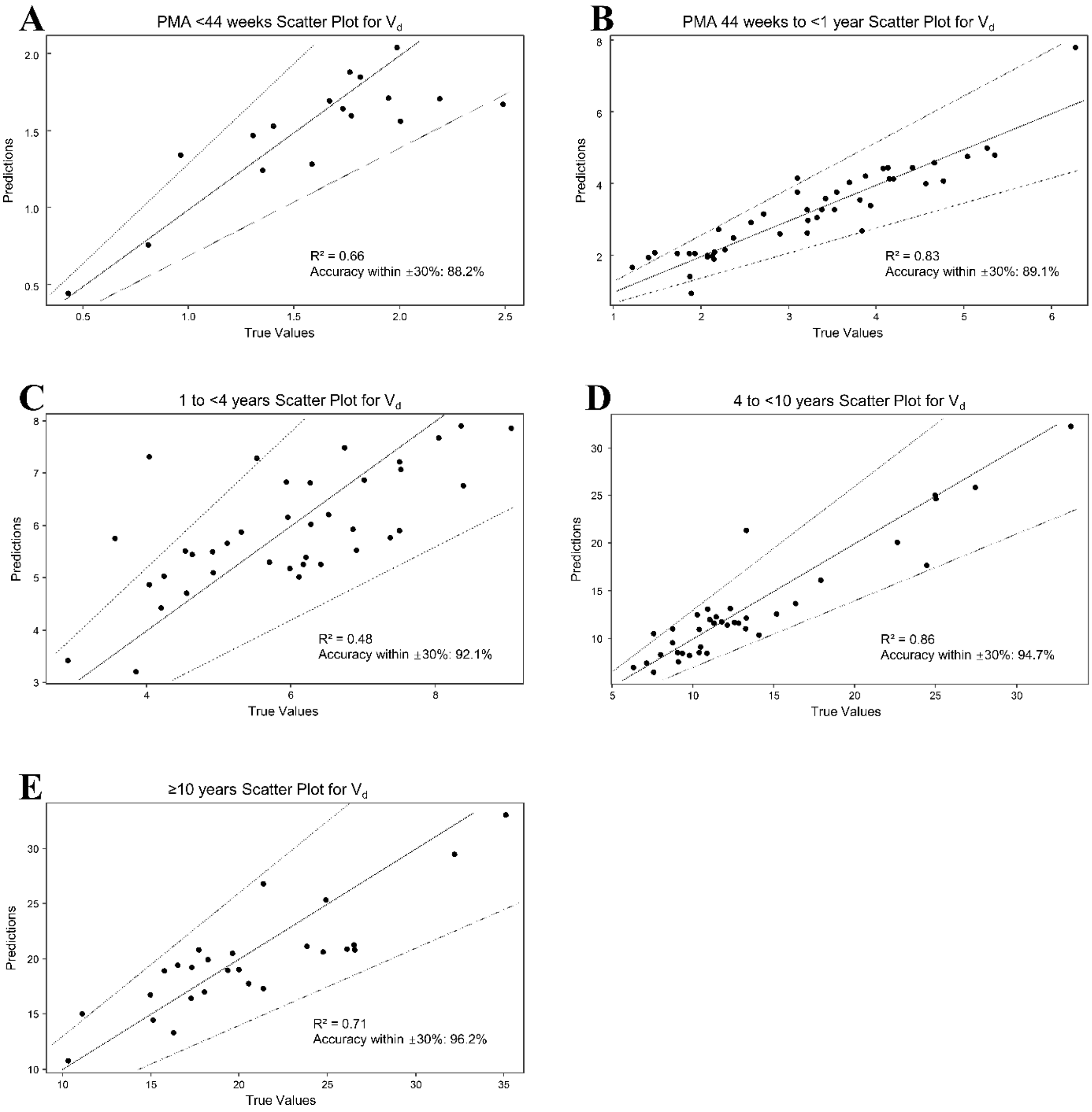

Figure S12. Scatter plots of V_d for different age subgroups based on the CatBoost model.

A) PMA <44 weeks, B) PMA ≥44 weeks to <1 year, C) 1 to <4 years, D) 4 to <10 years, and E) ≥10 years.

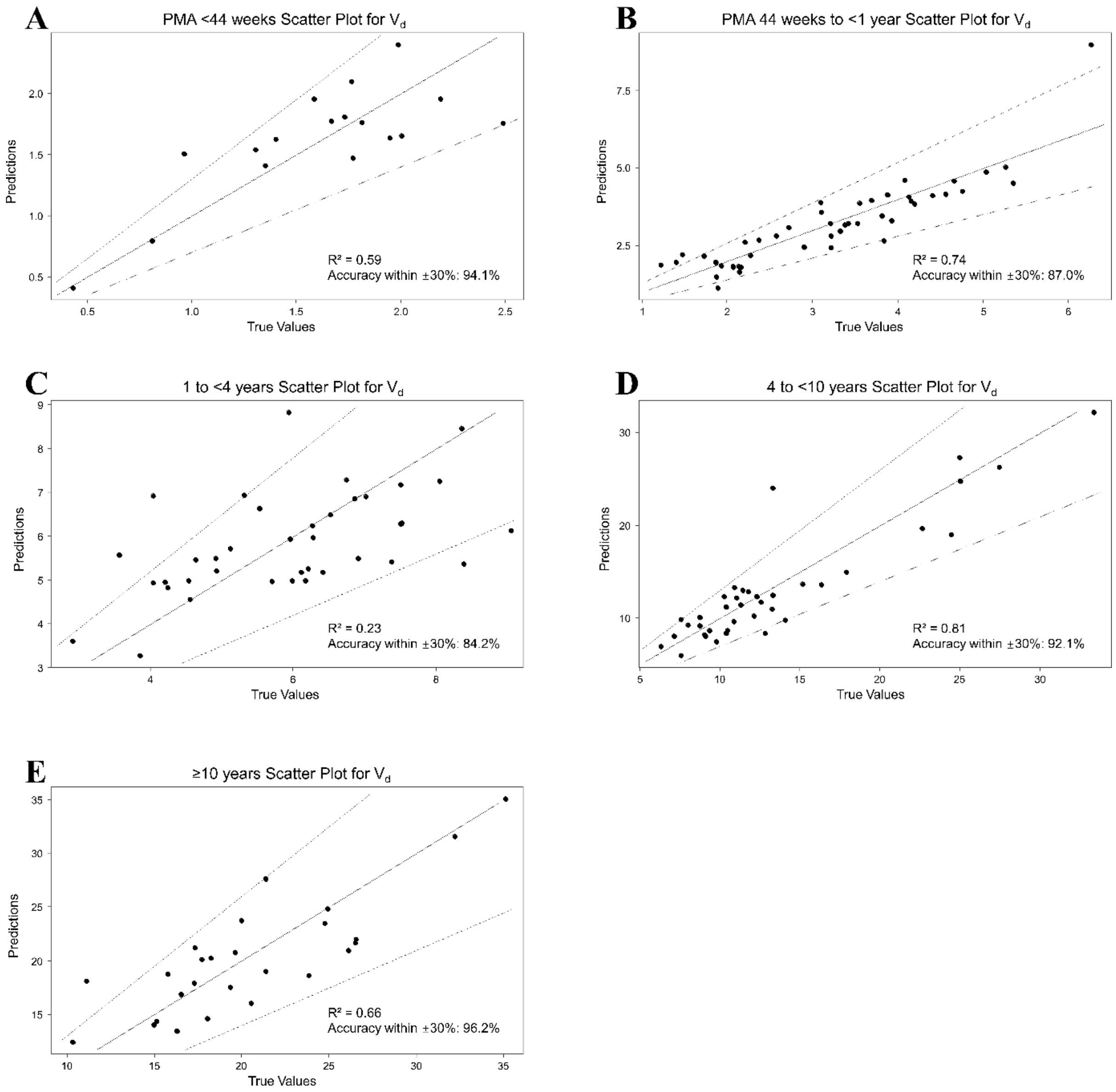

Figure S13. Scatter plots of V_d for different age subgroups based on the PPK model. A) PMA <44 weeks, B) PMA ≥44 weeks to <1 year, C) 1 to <4 years, D) 4 to <10 years, and E) ≥10 years.

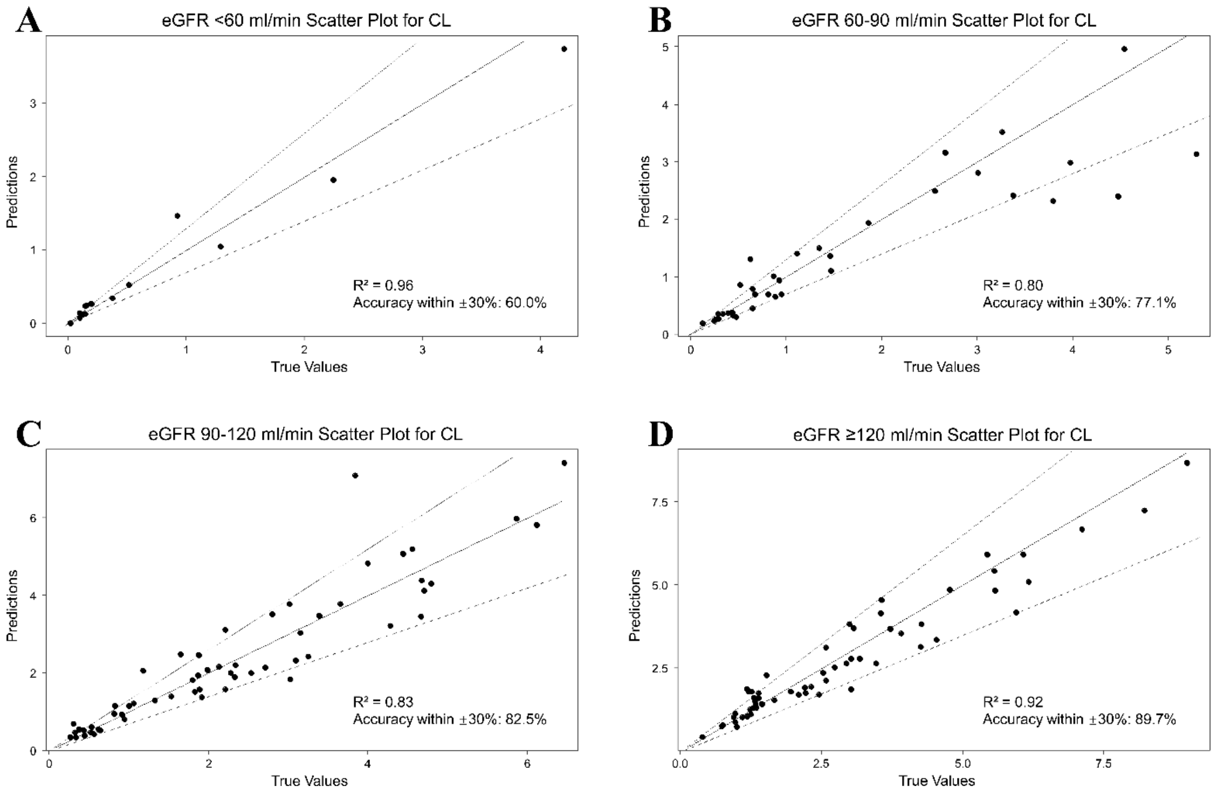

Figure S14. Scatter plots of CL for different renal function subgroups based on the CatBoost model. A) eGFR <60 ml/min, B) eGFR 60-90 ml/min, C) eGFR 90-120 ml/min, and D) eGFR ≥120 ml/min.

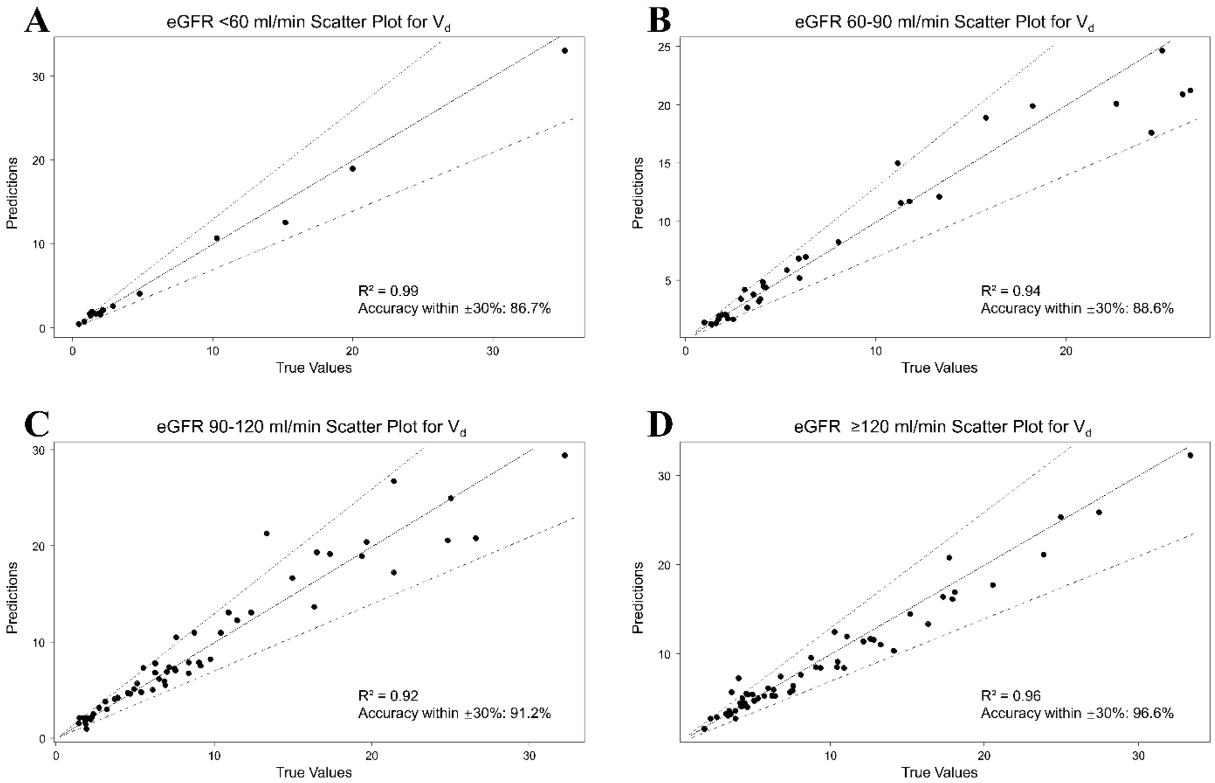

Figure S15. Scatter plots of CL for different renal function subgroups based on the PPK model. A) eGFR <60 ml/min, B) eGFR 60-90 ml/min, C) eGFR 90-120 ml/min, and D) eGFR ≥120 ml/min.

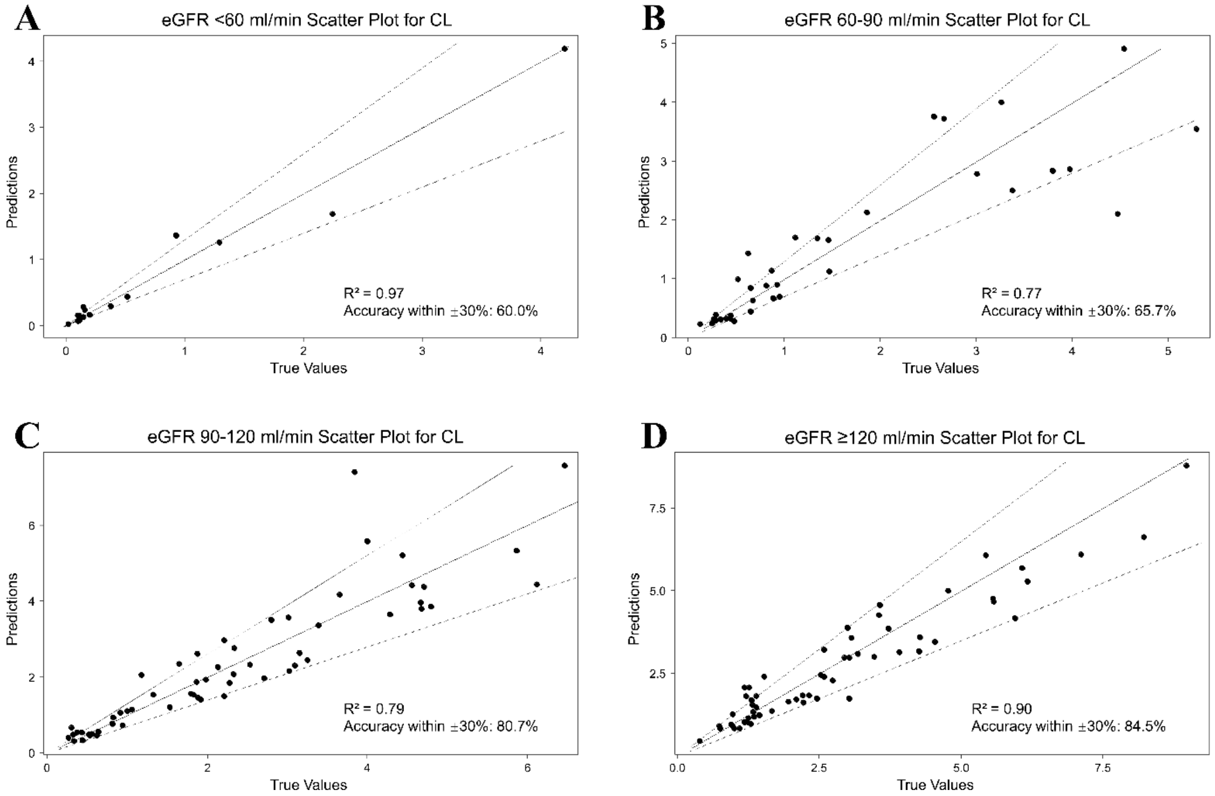

Figure S16. Scatter plots of V_d for different renal function subgroups based on the CatBoost model. A) eGFR <60 ml/min, B) eGFR 60-90 ml/min, C) eGFR 90-120 ml/min, and D) eGFR ≥120 ml/min.

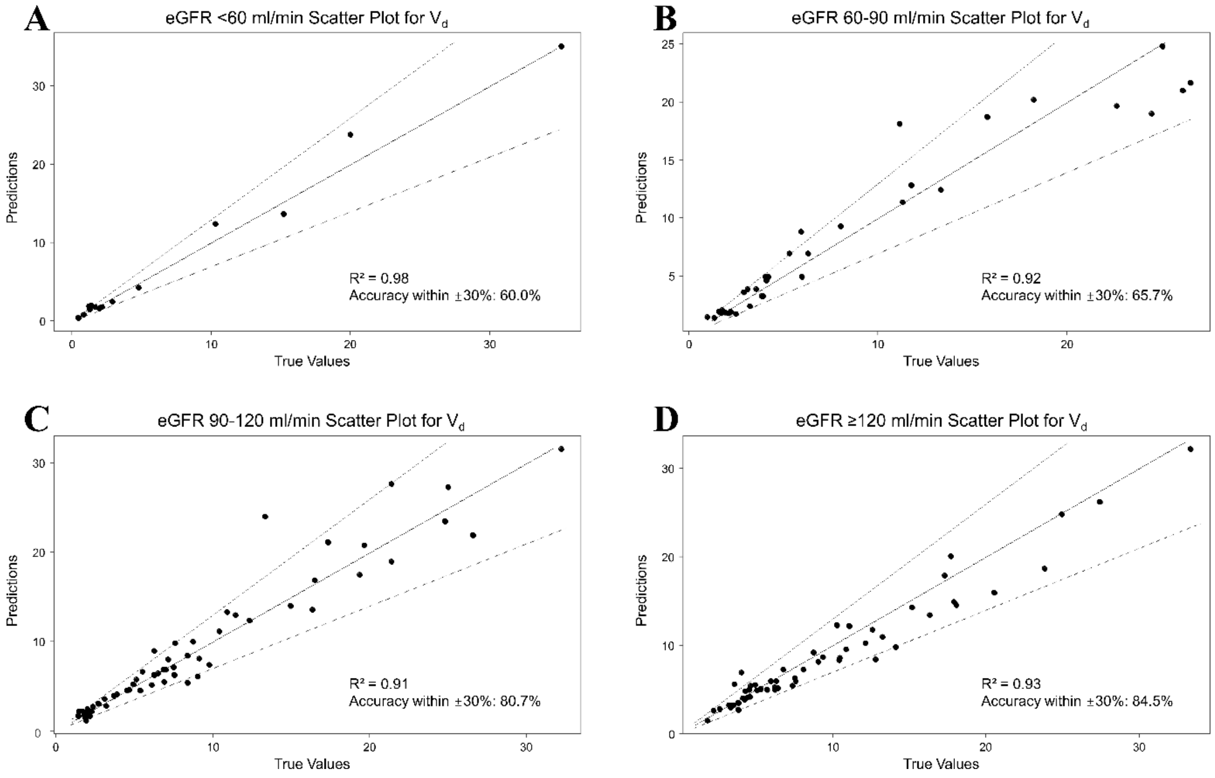

Figure S17. Scatter plots of V_d for different renal function subgroups based on the PPK model. A) eGFR <60 ml/min, B) eGFR 60-90 ml/min, C) eGFR 90-120 ml/min, and D) eGFR ≥120 ml/min.
